# Tetra-arylborate lipophilic anions as targeting groups†
†Electronic supplementary information (ESI) available: Author contributions, movies, synthetic procedures, NMR spectra and supplementary figure. See DOI: 10.1039/d0cc07924c


**DOI:** 10.1039/d0cc07924c

**Published:** 2021-02-18

**Authors:** Kishore K. Gaddale Devanna, Justyna M. Gawel, Tracy A. Prime, Filip Cvetko, Cristiane Benincá, Stuart T. Caldwell, Alexander Negoda, Andrew Harrison, Andrew M. James, Evgeny V. Pavlov, Michael P. Murphy, Richard C. Hartley

**Affiliations:** a School of Chemistry , University of Glasgow , Glasgow , G12 8QQ , UK . Email: Richard.Hartley@glasgow.ac.uk; b MRC Mitochondrial Biology Unit , Hills Road , University of Cambridge , CB2 0XY , UK . Email: mpm@mrc-mbu.cam.ac.uk; c Department of Physiology and Biophysics , Dalhousie University , Halifax , Nova Scotia , Canada; d New York University , College of Dentistry , Department of Molecular Pathobiology , 345 East 24th Street , New York , NY 10010 , USA; e Department of Medicine , University of Cambridge , Cambridge , UK

## Abstract

Tetraphenylborate (TPB) anions traverse membranes but are excluded from mitochondria by the membrane potential (Δ*ψ*). TPB-conjugates also distributed across membranes in response to Δ*ψ*, but surprisingly, they rapidly entered cells. They accumulated within lysosomes following endocystosis. This pH-independent targeting of lysosomes makes possible new classes of probe and bioactive molecules.

## Abstract 2

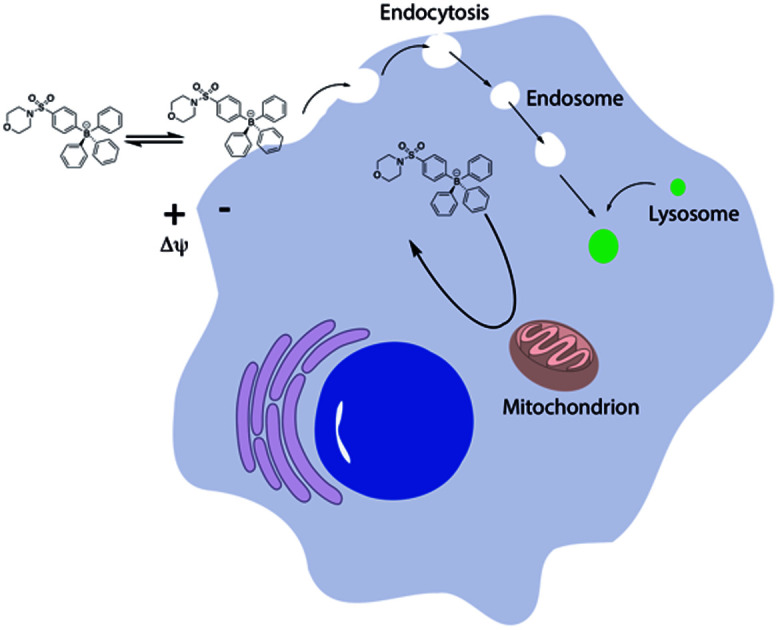
TPB lipophilic anions deliver cargoes to lysosomes and are excluded from mitochondria.

## 

The ability to direct molecules to an appropriate location within the cell facilitates development of bioactive and probe compounds. For example, molecules are targeted to the mitochondrial matrix by conjugation to the lipophilic triphenylphosphonium cation.^1^–^4^ The large hydrophobic surface area of the triphenylphosphonium group enables rapid crossing of biological membranes by lowering the activation energy for movement through the membrane core.^4^,^5^ Furthermore, the positive charge drives extensive accumulation within the mitochondrial matrix due to the large membrane potential (Δ*ψ* negative inside).^2^,^4^,^5^

To deliver molecules to other cell compartments in a similar way, we explored conjugation to lipophilic anions. Just as the archetypal lipophilic cation is tetraphenylphosphonium (TPP) the corresponding lipophilic anion is tetraphenylborate (TPB). These molecules have identical radii (4.2 Å) and, apart from charge, are similar.^6^,^7^ Both are water soluble, facilitating biological uses, although TPB has a more negative free energy of hydration.^8^ Lipophilic ions bind to a potential energy well on the membrane surface, before flipping, to the corresponding potential energy well on the other side of the membrane, traversing the activation energy barrier of the membrane core (Fig. 1).^6^,^7^,^9^ Hence, TPB rapidly permeates phospholipid bilayers^6^,^7^,^10^,^11^ and its distribution is determined by the Δ*ψ*,^12^ resulting in exclusion from the mitochondrial matrix.

**Fig. 1:**
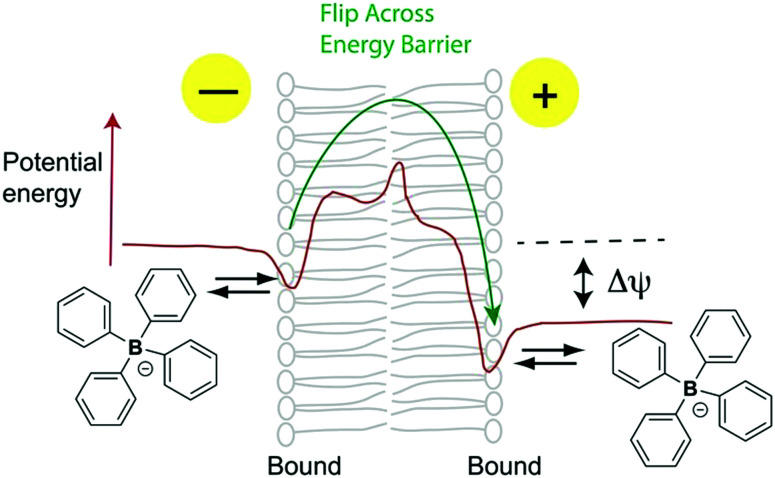
Membrane permeation by TPB lipophilic anions (adapted from ref. 9).

TPB anions have been widely used in analytical chemistry,^15^ catalysis^16^ and solid electrolytes,^17^ and as substrates for oxidative^18^,^19^ and Suzuki-type cross-coupling.^20^,^21^ Despite their similar structure to TPP, and their complementary response to Δ*ψ*, the use of TPB anions to deliver compounds to different sub-cellular compartments has not been explored. This may be because the Δ*ψ* across the plasma membrane, which is negative inside, would be expected to disfavour intracellular delivery. There has been only one exploration of lipophilic anion distribution within cells, by conjugation of the monocarborane (1-carba-*closo*-dodecaborate) lipophilic anion to a porphyrin.^13^,^14^ Surprisingly, the monocarborane conjugate was taken up by cells,^14^ although the mechanism was not investigated. Thus, we investigated whether TPB anions could be used to deliver compounds, despite their negative charge, within the cell.

We synthesized a universal TPB-conjugate precursor **5** that could be derivatized through amide coupling to carboxylic acids. The key synthetic step was the addition of three identical phenyl substituents to an aryltrifluoroborate (Scheme 1).^22^ As tetraarylborates with electron-rich substituents are prone to oxidation and protodeborylation,^23^ we incorporated an electron-withdrawing sulfonamide. To assess intracellular distribution and functional properties we made a minimal TPB derivative with the sulfonamide piperazine replaced with a morpholine (TPBM), to avoid N-protonation targeting acidic compartments;^24^,^25^ a TPB conjugated to the chromanol group of α-tocopherol as a generic cargo (TPBE); and two fluorescent probes, TPBCoumarin and TPBBODIPY, to allow imaging.

**Scheme 1:**
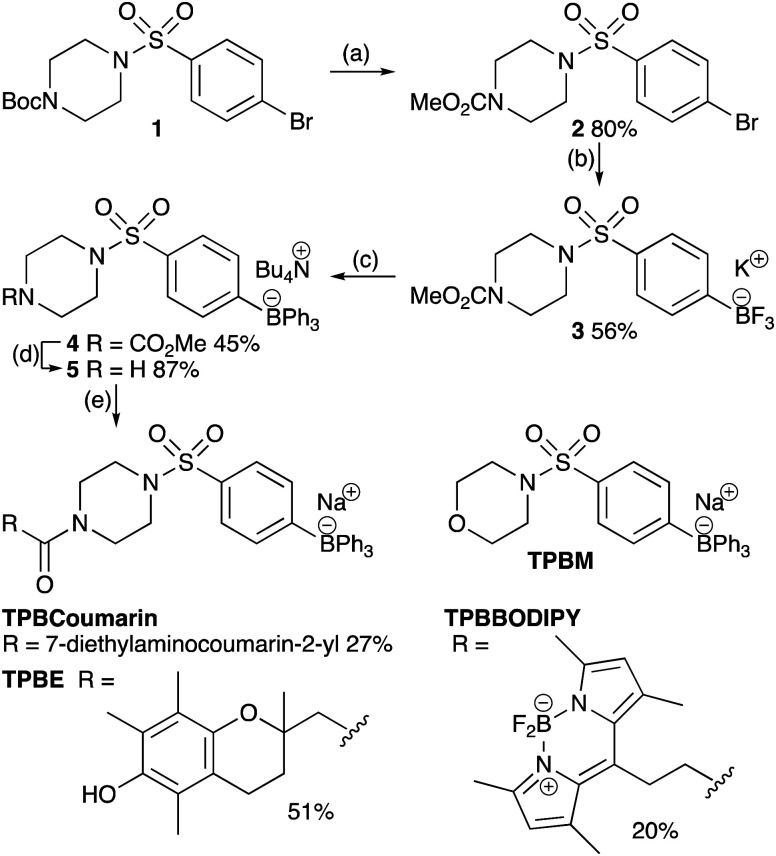
Synthesis of TPB-conjugates. Conditions: (a) (i) TFA-CH_2_Cl_2_, 0 °C – RT, 2 h (ii) ClCO_2_Me, 0 °C - RT, 3.5 h. (b) (i) [B(Pin)]_2_, Pd(dppf)Cl_2_, KOAc, DMSO, 70 °C, 18 h (ii) KF, MeCN–MeOH (1 : 1), RT, 1 min (iii) l-(+)-tartaric acid, THF, RT, 2 min (iv) MeCN 4 min (c) (i) 5 equiv. PhMgCl, THF, 0 °C, 30 min then reflux 16 h, (ii) Na_2_CO_3_(aq), RT, 1 h (iii) Bu_4_NBr, CH_2_Cl_2_, RT. (d) KOH, MeOH–H_2_O (2 : 1) reflux, 4 h. (e) (i) RCO_2_H, coupling agent (ii) ion exchange.

To see if TPB-conjugates could rapidly distribute across membranes in response to voltage we used a phospholipid black lipid membrane (BLM) system (Fig. 2). In agreement with previous observations,^26^ we detected increasing currents due to TPB crossing a BLM as a function of voltage (Fig. 2a). In the presence of a TPB gradient (1 μM : 10 μM) across a BLM the reversal potential was close to the theoretical Nernst equilibrium potential of 60 mV (Fig. 2a), demonstrating that the observed current is caused by TPB transfer across the bilayer. At higher TPB concentrations, but the same gradient, the current increased and the reversal potential shifted towards 0 mV, due to non-specific conductance caused by membrane disruption (Fig. 2b). TPBM disrupted the BLM at lower concentrations than TPB, leading to a larger non-specific conductance, hence the reversal potential for TPBM was considerably lower than 60 mV (Fig. 2c). The greater membrane disruption by TPBM compared to TPB is caused by its enhanced lipophilicity. Therefore, only low concentrations can be used and it was not possible to assess the membrane permeation of TPBM and TPBE directly. Instead, to estimate the amount of compound crossing the bilayer in response to a voltage we measured transient ionic currents induced by steps from 0 mV to defined voltages before/after addition of the compounds (Fig. 2d).

**Fig. 2:**
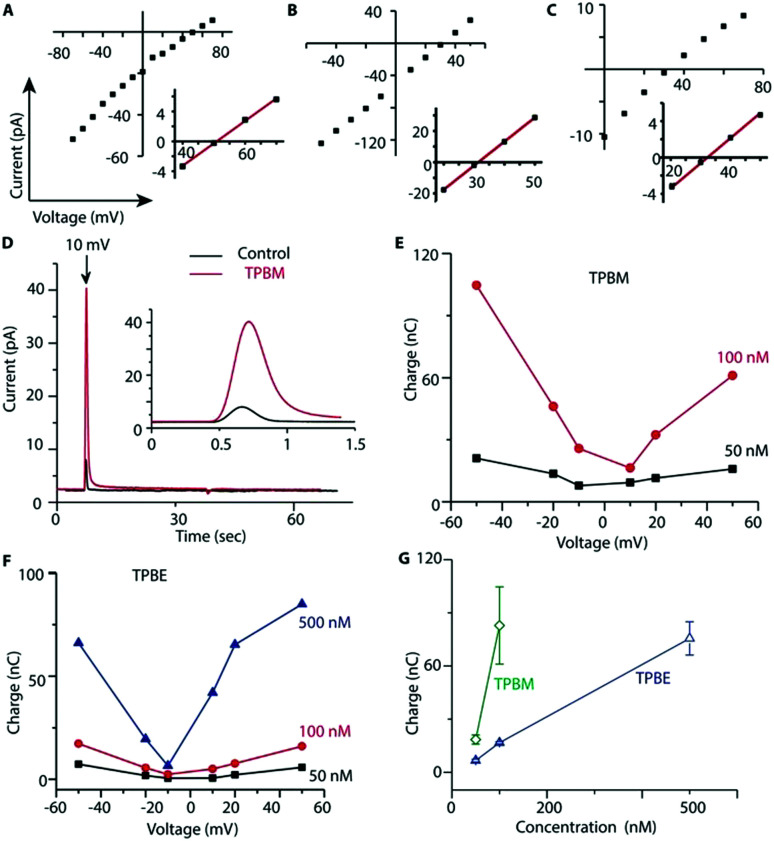
Transport of TPB-conjugates across a Black Lipid Membrane (BLM). Compounds were added to the both sides of a BLM to create a concentration gradient. Insets show expansions of the *x*-axis intersection that indicates reversal potential (*E*_rev_). (A) TPB gradient = 10 μM (*cis*)/1 μM (*trans*), *E*_rev_ ∼50 mV. (B) TPB gradient = 60 μM (*cis*)/10 μM (*trans*), *E*_rev_ ∼31 mV. (C) TPBM = 5 μM (*cis*)/0.5 μM (*trans*), *E*_rev_ ∼32.5 mV. (D) Current recorded at 10 mV ± 100 nM TPBM. Inset shows magnification of the region at voltage application. (E and F) Charge accumulated on BLM with different concentrations of TPBM and TPBE as a function of applied voltage. (G) Data from independent experiments at voltage jump +50 mV. *n* = 4–6 ± SEM.

In the absence of compounds the transient (capacitive) currents are determined by the membrane's dielectric properties. In the presence of a TPB-conjugates the transient currents are the sum of the capacitive current and that caused by anion redistribution.^27^ Charge transfer by the lipophilic anions crossing the bilayer, derived by subtracting the current in the absence of compound, depends on the compound concentration and the applied voltage (Fig. 2e and f). Combined data for a +50 mV voltage jump are shown in Fig. 2g. TPBM at 100 nM induced the largest charge flux, while higher concentrations caused non-specific conductance. The TPBE conductance was less (Fig. 2e and f). These results indicate that TPB-conjugates cross a BLM in a voltage-dependent manner.

We next assessed whether TPB-conjugates crossed biological membranes in response to a Δ*ψ* using sub-mitochondrial particles (SMPs), which are inverted mitochondrial inner membrane vesicles (Fig. 3a). Proton pumping by the respiratory chain generates a positive-inside Δ*ψ*, which should drive uptake of lipophilic anions^12^ (Fig. 3a).

**Fig. 3:**
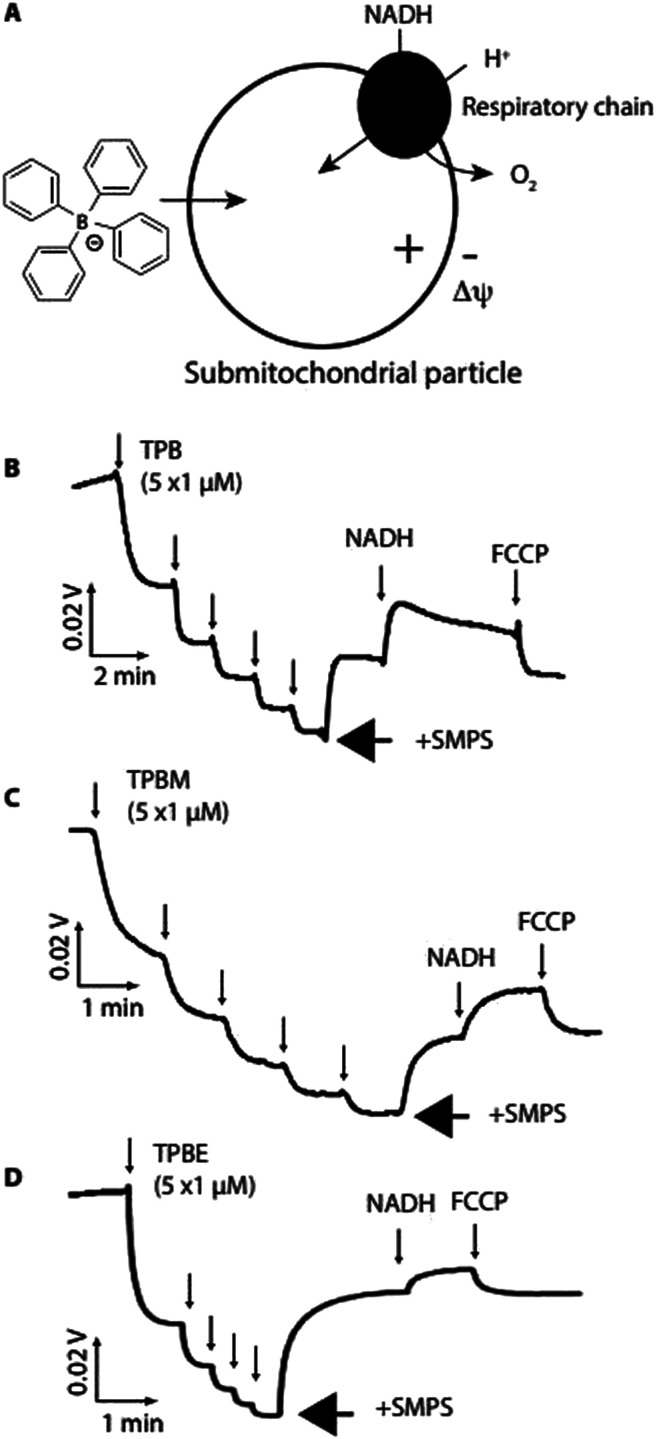
Uptake of TPB derivatives by submitochondrial particles (SMPs). (A), Upon energization with NADH proton pumping by the respiratory chain generates a Δ*ψ*, negative inside. (B–D), An ion-selective electrode (ISE) was calibrated (5 × 1 μM additions) then SMPs (0.2 mg protein per ml) were added followed by NADH (1 mM) to generate Δ*ψ* and subsequently FCCP (1 μM) to dissipate Δ*ψ*.

Using an ion-selective electrode (ISE) to measure TPB concentration^28^ showed its rapid uptake into SMPs upon induction of a Δ*ψ* by NADH, which was reversed by dissipating the Δ*ψ* with FCCP (Fig. 3b). Similarly, TPBM (Fig. 3c) and TPBE (Fig. 3d) also showed uptake into SMPs in response to Δ*ψ*. The greater hydrophobicity of TPBM and TPBE enhanced their membrane adsorption compared to TPB. Therefore, TPB-conjugates rapidly cross biological membranes in response to a Δ*ψ* and adsorb strongly to membranes.

Importantly, these attributes of TPB-conjugates are greatly enhanced over lipophilic cations due to the different interaction of lipophilic anions with biological membranes. This is primarily because of the large dipole potential from positive within the phospholipid bilayer core to negative at the surface^6^,^7^,^9^,^29^ which lowers the activation energy barrier for lipophilic anion transport, relative to cations, hence the membrane conductivity of TPB is ∼10^5^-fold greater than that for TPP.^6^,^7^,^9^–^11^ Similarly, the binding constant for TPB to the potential energy well near the membrane surface is ∼10^3^–10^4^ fold greater than for TPP.^6^,^7^,^9^,^29^ Addition of a hydrophobic linker/cargo increases this membrane adsorption further still.

We next assessed the uptake and distribution of TPB-conjugates using a fluorescent probe, TPBCoumarin, with C2C12 cells by confocal laser scanning microscopy (Fig. 4a and Movie 1, ESI†). Despite its negative charge, TPBCoumarin was rapidly taken up by cells showing punctate staining, as was TPBBODIPY (Fig. S1 and Movie 2, ESI†). Parallel staining with MitoTracker (Fig. 4b) showed TPBCoumarin was not colocalizing with mitochondria while LysoTracker (Fig. 4c) showed partial colocalization with lysosomes. This suggests the punctate staining upon uptake of TPBCoumarin is due to uptake by endocytosis with initial localization within endosomes which then fuse with lysosomes.^30^ Supporting this, uptake was greatly decreased by Pitstop 2 (Fig. 4d), which inhibits clathrin-mediated endocytosis.^31^,^32^ This shows there is rapid initial uptake by endocytosis followed by redistribution to lysosomes. Critically, there was no co-localization with mitochondria, consistent with their exclusion from the negatively-charged matrix. Cell toxicity of TPBE was also negligible below 10 μM (Fig. S2, ESI†).

**Fig. 4:**
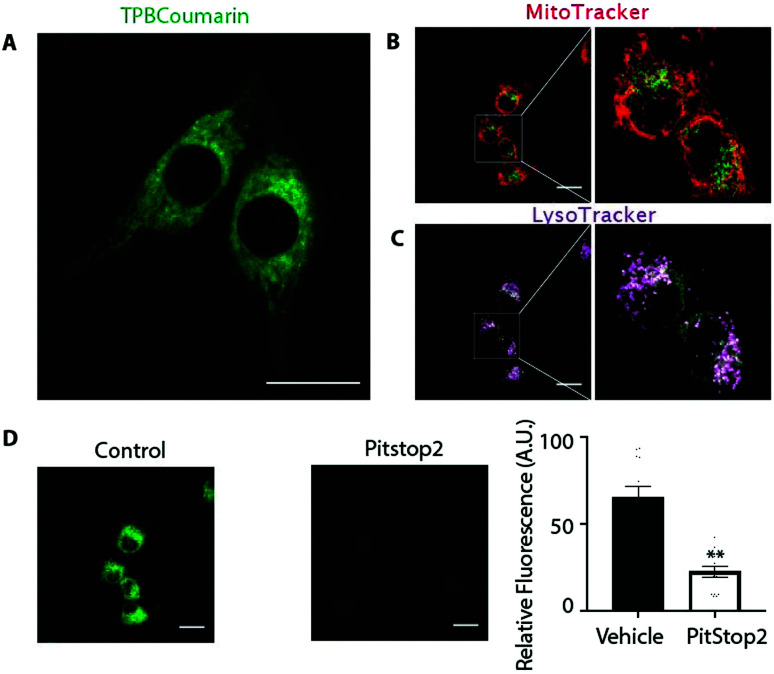
Cell distribution of TPBCoumarin. (A), C2C12 cells were incubated with 100 nM TPBCoumarin (green). Distribution imaged 5 min after addition. (B) MitoTracker (red) was compared with TPBCoumarin (green). (C) LysoTracker (magenta) compared with TPBCoumarin (green). Colocalization is shown in white. (D) Cells were incubated with 20 μM Pitstop2 for 30 min before addition of TPBCoumarin and compared with control. The bar chart shows mean ± SEM of 3 independent experiments. ** *p* <0.01, by Student's *t*-test. Scale bar = 20 μm.

Despite their negative charge, and the negative-inside Δ*ψ* across the plasma membrane, TPB display a novel intracellular distribution. Most lysosome targeting is achieved through ion-trapping by protonation of weak base in the acidic lysosomes.^24^,^25^ The TPBBODIPY conjugates accumulate in the same way as TPBCoumarin (Fig. 4 and Fig. S1, Movies 1, 2, ESI†), showing that TPB-conjugates do not require such as site. Both TPBCoumarin and TPBBODIPY showed similar distribution in both HeLa and Cos7 cells showing the unique distribution was not dependent on the cell type (Fig. S3, ESI†). Their rapid endocytic uptake is likely due to the strong binding of the TPB moiety to the potential energy well on the membrane surface. Since TPB-conjugates could rapidly permeate the phospholipid bilayers of SMPs, there is also likely to be some uptake into cells directly through the plasma membrane. However, this is disfavoured by the plasma membrane potential (30–60 mV, negative inside). Any TPB-conjugates that do enter may be directed to the lysosomes in response to the negative-inside potential across the lysosomal membrane.^33^ The lack of co-localization with mitochondria is consistent with the expected thousand-fold exclusion due to the large Δ*ψ* (150–180 mV, negative inside) across the mitochondrial inner membrane.

In summary, we have generated a new targeting group that directs small molecules to the endosomal and lysosomal compartments within the cell in a way that complements current targeting methods that employ ion-trapping of weak bases. By manipulating hydrophobicity and incorporating cleavable linkers and membrane impermeant moieties it will be possible to fine tune the location and kinetics of cell distribution of bioactive molecules. This approach provides new opportunities to selectively manipulate and report on cell processes and to give a better understanding of the role of the lysosome in autophagy^34^,^35^ and in diseases such as cancer.^36^,^37^

This work was supported by the Medical Research Council UK (MC_U105663142), a Wellcome Trust Investigator award (110159/A/15/Z) to MPM, by BBSRC (BB/I012826/1) and Wellcome Trust Investigator (110158/Z/15/Z) awards to RCH, a University of Glasgow funded studentship to J. G. and from the Wellcome Trust PhD programme in metabolic and cardiovascular diseases (RG88195) for FC.

## Conflicts of interest

There are no conflicts to declare.

## Supplementary Material

Supplementary information

Supplementary information

Supplementary movie

Supplementary movie

## References

[cit1] Murphy M. P., Hartley R. C. (2018). Nat. Rev. Drug Discovery.

[cit2] Smith R. A. J., Hartley R. C., Cocheme H. M., Murphy M. P. (2012). Trends Pharmacol. Sci..

[cit3] Yousif L. F., Stewart K. M., Kelley S. O. (2009). ChemBioChem.

[cit4] Zielonka J., Joseph J., Sikora A., Hardy M., Ouari O., Vasquez-Vivar J., Cheng G., Lopez M., Kalyanaraman B. (2017). Chem. Rev..

[cit5] Ross M. F., Kelso G. F., Blaikie F. H., James A. M., Cocheme A., Filipovska T., Da Ros T. R., Hurd R. A., Smith H. M., Murphy M. P. (2005). Biochemistry (Moscow).

[cit6] Flewelling R. F., Hubbell W. L. (1986). Biophys. J..

[cit7] Flewelling R. F., Hubbell W. L. (1986). Biophys. J..

[cit8] Scheu R., Rankin B. M., Chen Y., Jena K. C., Ben-Amotz D., Roke S. (2014). Angew. Chem., Int. Ed..

[cit9] Wang L. (2012). Annu. Rev. Biochem..

[cit10] Benz R. (1988). Biophys. J..

[cit11] Rokitskaya T. I., Luzhkov V. B., Korshunova G. A., Tashlitsky V. N., Antonenko Y. N. (2019). Phys. Chem. Chem. Phys..

[cit12] Grinius L. L., Jasaitis A. A., Kadziauskas Y. P., Liberman E. A., Skulachev V. P., Topali V. P., Tsofina L. M., Vladimirova M. A. (1970). Biochim. Biophys. Acta.

[cit13] Rokitskaya T. I., Zaitsev A. V., Ol'shevskaya V. A., Kalinin V. N., Moisenovich M. M., Agapov I. I., Antonenko Y. N. (2012). Biochemistry.

[cit14] Moisenovich M. M., Ol'shevskaya V. A., Rokitskaya T. I., Ramonova A. A., Nikitina R. G., Savchenko A. N., Tatarskiy Jr. V. V., Kaplan M. A., Kalinin V. N., Kotova E. A., Uvarov O. V., Agapov I. I., Antonenko Y. N., Shtil A. A. (2010). PLoS One.

[cit15] Flaschka H., Barnard A. J. (1960). Adv. Anal. Chem. Instrum..

[cit16] Riddlestone I. M., Kraft A., Schaefer J., Krossing I. (2018). Angew. Chem., Int. Ed..

[cit17] Van Humbeck J. F., Aubrey M. L., Alsbaiee A., Ameloot R., Coates G. W., Dichtel W. R., Long J. R. (2015). Chem. Sci..

[cit18] Gerleve C., Studer A. (2020). Angew. Chem., Int. Ed..

[cit19] Music A., Baumann A. N., Spiess P., Plantefol A., Jagau T. C., Didier D. (2020). J. Am. Chem. Soc..

[cit20] Hussain I., Capricho J., Yawer M. A. (2016). Adv. Synth. Catal..

[cit21] Vasu D., Yorimitsu H., Osuka A. (2015). Synthesis.

[cit22] Franzke A., Pfaltz A. (2008). Synthesis.

[cit23] Nishida H., Takada N., Yoshimura M., Sonoda T., Kobayashi H. (1984). Bull. Chem. Soc. Jpn..

[cit24] Zhu H., Fan J., Du J., Peng X. (2016). Acc. Chem. Res..

[cit25] Xu W., Zeng Z., Jiang J.-H., Chang Y.-T., Yuan L. (2016). Angew. Chem., Int. Ed..

[cit26] Rokitskaya T. I., Klishin S. S., Severina I. I., Skulachev V. P., Antonenko Y. N. (2008). J. Membr. Biol..

[cit27] Armstrong C. M., Bezanilla F. (1977). J. Gen. Physiol..

[cit28] Shoukry A. F., Badawy S. S., Issa Y. M. (1987). Anal. Chem..

[cit29] Honig B. H., Hubbell W. L., Flewelling R. F. (1986). Annu. Rev. Biophys. Biophys. Chem..

[cit30] Doherty G. J., McMahon H. T. (2009). Annu. Rev. Biochem..

[cit31] Macia E., Ehrlich M., Massol R., Boucrot E., Brunner C., Kirchhausen T. (2006). Dev. Cell.

[cit32] von Kleist L., Stahlschmidt W., Bulut H., Gromova K., Puchkov D., Robertson M. J., MacGregor K. A., Tomilin N., Pechstein A., Chau N., Chircop M., Sakoff J., von Kries J. P., Saenger W., Krausslich H. G., Shupliakov O., Robinson P. J., McCluskey A., Haucke V. (2011). Cell.

[cit33] Xu H., Ren D. (2015). Annu. Rev. Physiol..

[cit34] Yim W. W.-Y., Mizushima N. (2020). Cell Discovery.

[cit35] Wong Y.-K., Zhang J., Hua Z.-C., Lin Q., Shen H.-M., Wang J. (2017). Autophagy.

[cit36] Geisslinger F., Müller M., Vollmar A. M., Bartel K. (2020). Front. Radiat. Oncol..

[cit37] Levine B., Kroemer G. (2008). Cell.

