# Supplementary material for: Tetra-arylborate lipophilic anions as targeting groups
Source: Chem Commun (Camb). 2021 Feb 18;57(25):3147–50. doi: 10.1039/d0cc07924c (PMC8062962; doi:10.1039/d0cc07924c)
Supplement: Supplementary file 1 [file CC-057-D0CC07924C-s001.pdf]

Electronic Supplementary Material (ESI) for Chemical Communications.
This journal is © The Royal Society of Chemistry 2021

Supporting information 1 for

Tetra-Arylborate Lipophilic Anions as Targeting Groups

Kishore Kumar Gaddale Devanna[1], Justyna M. Gawel[1], Tracy A. Prime[2], Filip Cvetko[2], Cristiane Benincá[2], Stuart T. Caldwell[1], Alexander Negoda[3], Andrew Harrison[2], Andrew M. James[2], Evgeny V. Pavlov[4], Michael P. Murphy[2,5,*] and Richard C. Hartley[1,*]

[1]School of Chemistry, University of Glasgow, Glasgow, G12 8TA, UK.

[2]MRC Mitochondrial Biology Unit, Hills Road, Cambridge CB2 0XY, UK.

[3]Department of Physiology and Biophysics, Dalhousie University, Halifax, Nova Scotia, Canada.

[4]New York University, College of Dentistry, Department of Molecular Pathobiology, 345 East 24th Street, New York, NY 10010, USA

[5]Department of Medicine, University of Cambridge, Cambridge, UK.

*Correspondence: Richard.Hartley@glasgow.ac.uk mpm@mrc-mbu.cam.ac.uk

Contents of Supplementary Information 1

1	Chemical Methods	
	1.1 Overview of Synthesis	S2
	1.2 General Information on Synthetic Procedures	S3
	1.3 Synthetic Procedures	S3
2	Black Lipid Membrane Experiments	S11
3	Submitochondrial Particle Experiments	S12
4	Cell Culture Experiments	S13
5	Confocal Microscopy	S14
6	Author Contributions	S15
7	References	S16
Figure S1: Cell distribution of TPBBODIPY in C2C12 cells		S17
Figure S2: Cell toxicity of TPBE		S18
Figure S3: Cell distribution of TPBBODIPY and TPBCoumarin in HeLa and Cos7 cells		S19
Figure S4: Fluorescence emission of TPBBODIPY and TPBCoumarin		S20
Movie Legends		S20

Supplementary Information 2 (separate file): NMR spectra

1. Chemical Methods

1.1 Overview of Synthesis

The general synthetic strategy to make the TPB compounds involved preparing TPB-amine **5** as a universal precursor TPB head group, which could then be coupled with a range of carboxylic acids to attach the desired cargo (Scheme 1). We followed a similar approach to Franzke and Pfaltz in the construction of tetraarylborates with one aryl group differing from the other three [1-2]. The sulfonamide precursor **1**, prepared from commercially available *N*-Boc piperidine and 4-bromosulfonyl chloride, was converted into methyl carbamate derivative **2** so that the protecting group could be removed using base rather than acid once the borate was installed. Miyaura borylation[3] was followed by conversion to the potassium aryltrifluoroborate salt **3** under the conditions of Lennox and Lloyd-Jones[4], with further removal of the pinacol side product with 50% aqueous MeOH and repeated evaporation-dissolution cycles[5]. Reaction with excess phenylmagnesium chloride completed the tetraarylborate head group and ion exchange to the tetrabutylammonium salt **4** ensured good solubility in organic solvents to assist purification. Removal of the carbamate protecting group then gave the TPB head group **5** bearing an amino group for attachment of any cargo. A carboxylic acid derivative of the chromanol group of α-tocopherol, 2-(6'-hydroxy-2',5',7',8'-tetramethylchroman-2'-yl)acetic acid, was prepared by the route of Scott *et al.*[6] and coupled with TPB-amine **5** to give TPBE using HBTU. The BODIPY carboxylic acid precursor, 3-(4,4-Difluoro-1,3,5,7-tetramethyl-4-bora-3a,4a-diaza-*s*-indacene-8-yl)-propionic acid, was prepared by the method of Thivierge et al.[7] and was coupled with amine **5** to give TPBBODIPY. The coumarin carboxylic acid was synthesised by the method of Tateishi et al[8] and was coupled to amine **5** to give TPBCoumarin. TPBM was prepared in a similar way to amine **5**, but using morpholine instead of *N*-Boc piperidine. Stock solutions of compounds were made up in ethanol, flushed with argon, and stored as aliquots at -20 °C before use. While developing the TPB compounds we also generated a number of other tetraphenylborate head groups that were less biologically effective that the sulfonamide derivative of TPB. The synthesis and characterisation of these will be described elsewhere.

1.2 General Information on Synthetic Procedures

All reactions under an inert atmosphere were carried out using oven-dried glassware and solvents were added *via* syringe. Reagents were obtained from commercial suppliers and used without further purification. Dry solvents were collected from a Puresolv solvent purification system or obtained from commercial suppliers. ^{1}H, ^{13}C, ^{11}B, and ^{19}F NMR spectra were obtained on a Bruker AVIII/400 spectrometer operating at 400, 101, 128, 377 and 162 MHz, respectively or a Bruker AVIII/500 operating at 500, 126, 160, 470 and 162 MHz, respectively. All coupling constants were measured in Hertz. Signals for carbon atoms attached to boron were not always observable in the ^{13}C NMR spectra, but where they were ^{11}B-^{13}C coupling contants were consistent with *J* values for related compounds [9]. Deuterated solvents contained trimethylsilane (TMS) as a reference compound. DEPT was used to assign the signals in ^{13}C NMR spectra. Mass spectra (MS) were recorded on a Jeol JMS700 (MStation) spectrometer for EI and CI or Bruker Microtof-q for ESI. A Shimadzu FTIR-8400S spectrometer was used to obtain infrared (IR) spectra. Melting points were determined on a Reichert platform melting point apparatus. Microwave reactions were conducted using a CEM Discover TM Synthesis Unit (CEM Corp., Matthews, NC) and performed in glass vessels (capacity 10 mL) sealed with a septum. Purification of products was carried out by crystallization or column chromatography using silica gel, 70 – 230 mesh or using a Biotage Isolera one automated system. AA'BB' signals for *para*-substituted aryl units are reported as doublets corresponding to $J_{AB} + J_{AA'}$.

1.3 Synthetic Procedures

***Tert*-butyl 4-(4’-bromophenylsulfonyl)piperazine-1-carboxylate 1**

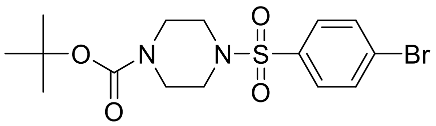

Piperazine-1-carboxylic acid *tert*-butyl ester (12.0 g, 64.4 mmol, 1 eq.) was dissolved in dry CH_2Cl_2 (400 mL) and DIPEA (22.4 mL, 129 mmol, 2 eq.) was added at 0 °C under an argon atmosphere. 4-Bromobenzenesulfonyl chloride (18.1 g, 70.9 mmol, 1.1 eq.) was added slowly portionwise to this stirred solution. The reaction mixture was stirred at 0 °C for 30 min and after that time was allowed to warm up to RT and stirred for 19 h. The reaction mixture was quenched with sat. aq. $NaHCO_3$ (400 mL) and extracted with CH_2Cl_2 (3 × 300 mL). The combined organics were dried over Na_2SO_4, filtered and concentrated under reduced pressure

to give *tert*-butyl ester **1** as a solid (26.0 g, 100%). δ_H (500 MHz, DMSO-d_6): 7.87 (2H, d, J=8.6 Hz, H-2', H-6'), 7.66 (2H, d, J=8.6 Hz, H-3', H-5'), 3.39 (4H, t, J=5.0 Hz, 2 × CH_2NCO), 2.87 (4H, t=5.0 Hz, 2 × CH_2NSO_2), 1.34 (9H, s, CH_3). δ_C (126 MHz, DMSO-d_6): 153.32 (C), 134.11 (C), 132.58 (CH), 129.51 (CH), 127.43 (C), 79.31 (C), 45.63 (2 × CH_2), 27.90 (CH_3). Spectroscopic data in accordance with literature.[10]

Methyl 4-(4'-bromophenylsulfonyl)piperazine-1-carboxylate 2

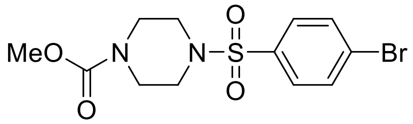

Tert-butyl ester **1** (26.7 g, 66.1 mmol, 1 eq.) was dissolved in dry CH_2Cl_2 (160 mL). TFA (106 mL, 1.45 mol, 22 eq.) was added slowly to this stirred solution at 0 °C under an argon atmosphere. The reaction mixture was stirred at 0 °C for 30 min and after that time was allowed to warm up to RT and stirred for 75 min. The solvent and excess TFA were removed under reduced pressure. The crude product was poured into Et_2O (1.0 L) and precipitated to give the corresponding trifluoroacetate salt as a solid (27.6 g, 100%). The ether layer was decanted off. The residual solvent was removed from the solid under high vacuum. The resulting trifluoroacetate salt was used without further purification. Methylchloroformate (5.60 mL, 72.6 mmol, 1.1 eq.) was added slowly to a stirred cooled solution (0 °C) of trifluoroacetate salt (27.6 g, 66.0 mmol, 1 eq.) and Et_3N (27.5 mL, 198 mmol, 3 eq.) in dry CH_2Cl_2 (200 mL) under an argon atmosphere. The reaction mixture was stirred at 0 °C for 30 min and after that time was allowed to warm up to RT and stirred for 3 h. The reaction mixture was quenched with 1 M HCl (600 mL) and extracted with CH_2Cl_2 (3 × 300 mL). The combined organics were dried over Na_2SO_4, filtered and concentrated under reduced pressure to give the carboxylate **2** as a solid (19.1 g, 80%) sufficiently pure for the next step. Mp: 120 - 122 °C. ν_{max} (ATR): 2964 (C-H), 2862 (C-H), 1699 (C=O). δ_H (500 MHz, DMSO-d_6): 7.86 (2H, d, J=8.6 Hz, H-2', H-6'), 7.66 (2H, d, J=8.6 Hz, H-3', H-5'), 3.54 (3H, s, CO_2CH_3), 3.44 (4H, t, J=5.0 Hz, 2 × CH_2NCO), 2.90 (4H, J=5.0 Hz, 2 × CH_2NSO_2). δ_C (126 MHz, DMSO-d_6): 154.72 (C), 134.19 (C), 132.60 (CH), 129.49 (CH), 127.45 (C), 52.49 (CH_3), 45.50 (CH_2), 42.69 (CH_2). HRMS (ESI^+): 384.9828. $C_{12}H_{15}N_2NaSO_4{}^{79}Br$ requires $[M+Na]^+$, 384.9820.

Potassium {4-[4'-(methoxycarbonyl)piperazin-1'-ylsulfonyl]phenyl}trifluoroborate 3

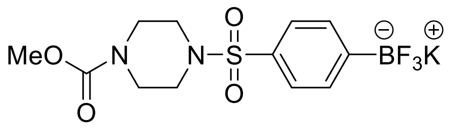

Ester **2** (5.0 g, 14 mmol, 1 eq.), $B(pin)_2$ (4.6 g, 18 mmol, 1.3 eq.), $Pd(dppf)Cl_2$ (500 mg, 0.700 mmol, 5 mol%) and KOAc (2.05 g, 20.7 mmol, 1.5 eq.) were combined together and the flask flushed with argon. The mixture was dissolved in dry DMSO (50 mL, previously degassed). The reaction mixture was stirred at 70 °C for 18 h under an argon atmosphere. The reaction mixture was allowed to cool down and extracted with EtOAc. The organic layer was washed with H_2O (1.5 L), brine (100 mL), dried over Na_2SO_4, filtered and concentrated under reduced pressure. The crude product was filtered through cellite and crystallized from MeCN to give pure pinacol ester intermediate as a beige solid (3.37 g, 60%). Pinacol ester (3.37 g, 8.19 mmol, 1 eq.) was suspended in MeOH (17 mL) and H_2O (17 mL). KF (1.87 g in 4.3 mL of H_2O) was added and the reaction mixture was stirred until complete dissolution of the boronic ester (1 min). L-(+)-tartaric acid (2.51 g, 16.8 mmol, 2.05 eq.) was dissolved in THF (13 mL) (gentle heat was required) and added dropwise to the rapidly stirring biphasic mixture over a period of 1 min, as a white precipitate formed. The reaction was stirred for 2 min, diluted with MeCN (24 mL) and stirred for a further 2 min before being diluted again with MeCN (7.4 mL) and filtered. The flask and the filter cake were rinsed with further portions of MeCN (3 × 40 mL) and then the combined filtrates were concentrated under reduced pressure. The solid residue was redissolved in 50% aq. MeOH (30 mL) and all volatile materials were removed under reduced pressure. Evaporation-dissolution cycles were repeated until ^{1}H NMR analysis of an aliquot of the reaction mixture showed no signal of pinacol [δ_H 1.14 (s, 12 H) ppm in CD_3CN]. The solid residue was dried by azeotropic removal of H_2O with toluene to give pure trifluoroboronate salt **3** as a beige solid (3.03 g, 56%). Mp: 270 - 272 °C. ν_{max} (ATR): 2986 (C-H), 1693 (C=O). δ_H (400 MHz, DMSO-d$_6$): 7.55 (2H, d, J=8.0 Hz, H-2, H-6), 7.45 (2H, d, J=8.0 Hz, H-3, H-5), 3.53 (3H, s, CO_2CH_3), 3.42 (4H, t, J=4.0 Hz, 2 × CH_2NCO), 2.80 (4H, J=4.0 Hz, 2 × CH_2NSO_2). δ_C (126 MHz, DMSO-d$_6$): 154.76 (C), 131.89 (CH), 131.16 (C), 125.56 (CH), 52.48 (CH_3), 45.65 (CH_2), 42.72 (CH_2). δ_F (470 MHz, DMSO-d$_6$): -140.16. LRMS (ESI$^-$): 352 [M$^-$ (borate anion, ^{11}B and ^{13}C), 14%], 351 [M$^-$ (borate anion, ^{11}B and ^{10}B plus ^{13}C), 100], 350 [M$^-$ (borate anion, ^{10}B), 24]. HRMS: 350.0835. $C_{12}H_{15}F_3N_2O_4S^{10}B$ requires [M$^-$ (borate anion)], 350.0839.

Tetrabutylammonium{4-[4'-(methoxycarbonyl)piperazin-1'ylsulfonyl]phenyl}triphenylborate 4

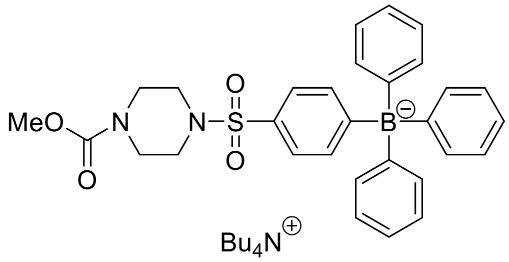

PhMgCl (3.2 mL, 6.4 mmol, 5 eq.) was added slowly dropwise to a stirred cooled solution (0 °C) of potassium trifluoroboronate salt **3** (500 mg, 1.28 mmol, 1 eq.) in dry THF (5 mL) under an argon atmosphere and stirred for 30 min. The reaction mixture was allowed to warm up to RT and heated under reflux for 16 h under an argon atmosphere. The reaction mixture was allowed to cool down to RT and was added slowly into an aqueous solution of Na_2CO_3 (3.31 g in 67 mL of H_2O). The reaction mixture was stirred vigorously for 60 min at RT and extracted with EtOAc. The organic layer was washed with H_2O and brine, dried over Na_2SO_4, filtered and concentrated under reduced pressure to give a crude product as an orange oil (732 mg). The crude product was dissolved in dry CH_2Cl_2 (10 mL) and tetrabutylammonium bromide (490 mg, 1.54 mmol, 1.2 eq.) dissolved in dry CH_2Cl_2 (7 mL) and was added slowly to this stirring solution. The reaction mixture was stirred for 30 min at RT, filtered and the filtrate was concentrated under reduced pressure to give a crude product as an orange oil (990 mg). Column chromatography [SiO_2, hexane:EtOAc (1:1→0:1)] gave tetrabutylammonium salt **4** as a white foam (441 mg, 45%). R_f [SiO_2, EtOAc]: 0.1. ν_{max} (ATR): 2962 (C-H), 1701 (C=O). δ_H (400 MHz, DMSO-d_6): 7.42 (2H, d, J=7.7 Hz, H-3, H-5), 7.31-7.28 (2H, m, H-2, H-6), 7.12 (6H, broad s, H-2", H-6"), 6.95 (6H, t, J=7.4 Hz, H-3", H-5"), 6.82 (3H, t, J=7.2 Hz, H-4"), 3.56 (3H, s, CO_2CH_3), 3.45-3.40 (4H, m, H_2-3', H_2-5'), 3.20-3.12 (8H, m, 4 × CH_2N), 2.83-2.79 (4H, m, H_2-2', H_2-6'), 1.61-1.50 (8H, m, 4 × CH_2), 1.35-1.25 (8H, m, 4 × CH_2), 0.93 (12H, t, J=7.3 Hz, 4 × CH_3). δ_C (126 MHz, DMSO-d_6): 154.80, 135.67, 135.31, 127.72, 125.56, 124.37, 121.93, 57.47, 52.44, 45.69, 42.62, 23.02, 19.18, 13.47. δ_B (128 MHz, DMSO-d_6): -6.53. LRMS (ESI$^-$): 525 [M^- (borate anion, ^{11}B and ^{10}B plus ^{13}C), 100%]. HRMS (ESI$^-$): 524.2061. $C_{30}H_{30}N_2O_4S^{10}B$ requires [M^- (borate anion)], 524.2043.

Tetrabutylammonium [4-(piperazin-1'-ylsulfonyl)phenyl]triphenylborate 5

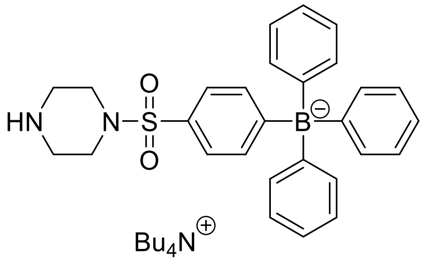

KOH (1.26 g, 22.9 mmol, 14 eq.) was added to a stirring solution of tetrabutylammonium salt **4** (1.26 g, 1.64 mmol, 1 eq.) in MeOH (9.0 mL) and H_2O (4.5 mL). The reaction mixture was refluxed for 48 h. The reaction mixture was allowed to cool down and the precipitate was collected by filtration. The solid was washed with cold MeOH and residual solvent removed under reduced pressure to give amine **5** as a solid (1.02 g, 87%). Mp: 132-134 °C. ν_{max} (ATR): 3576 (NH), 2931 (C-H), 1572 ($C_{Ar} = C_{Ar}$). δ_H (400 MHz, DMSO-d$_6$): 7.42 (2H, broad d, J=7.5 Hz, H-2, H-6), 7.28 (2H, d, J=8.0 Hz, H-3, H-5), 7.14 (6H, broad s, H-2", H-6"), 6.96 (6H, t, J=7.5 Hz, H-3", H-5"), 6.83 (3H, t, J=6.6 Hz, H-4"), 3.19-3.12 (8H, m, 4 × CH_2N), 2.85-2.65 (8H, m, H-2', H-3', H-5', H-6'), 1.60-1.52 (8H, m, 4 × CH_2), 1.35-1.27 (8H, m, 4 × CH_2), 0.93 (12H, t, J=7.4 Hz, 4 × CH_3). δ_C (126 MHz, DMSO-d$_6$): 161.98 (q, *J* (C-^{11}B) = 47.8 Hz, C-B), 135.58 (CH), 135.33 (CH), 127.80 (C), 125.77 (CH), 124.48 (CH), 121.92 (CH), 57.50 (CH_2), 49.77 (CH_2), 45.86 (CH_2), 23.02 (CH_2), 19.18 (CH_2), 13.46 (CH_3). δ_B (128 MHz, DMSO-d$_6$): -6.65. LRMS (ESI$^-$): 468 [M$^-$ (borate anion, ^{11}B and ^{13}C), 29%], 467 [M$^-$ (borate anion, ^{11}B and ^{10}B plus ^{13}C), 100], 466 [M$^-$ (borate anion, ^{10}B), 19]. HRMS: 466.1984. $C_{28}H_{28}N_2O_2S^{10}B$ requires [M$^-$ (borate anion)], 466.2006.

Tetrabutylammonium (4-{4'-[2"-(6'''-hydroxy-2''',5''',7''',8'''-tetramethylchroman-2'''-yl)acetyl]piperazin-1'-ylsulfonyl}phenyl)triphenylborate, TPBE (tetrabutylammonium salt)

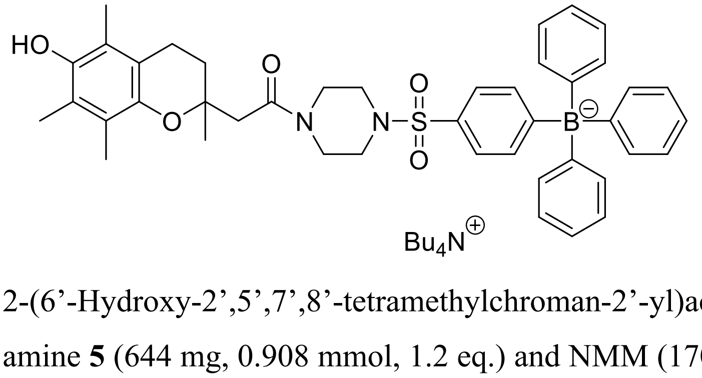

2-(6'-Hydroxy-2',5',7',8'-tetramethylchroman-2'-yl)acetic acid [6] 200 mg, 0.740 mmol, 1 eq.), amine **5** (644 mg, 0.908 mmol, 1.2 eq.) and NMM (170 µL, 1.48 mmol, 2 eq.) were dissolved in dry DMF (10 mL). HBTU (314 mg, 0.814 mmol, 1.1 eq.) was added and the reaction mixture

was stirred for 24 h at RT under an argon atmosphere. After that time H_2O (4 mL) was added and the reaction mixture was stirred for 10 min. A small amount of orange precipitate was observed. The whole reaction mixture was extracted with CH_2Cl_2 and the organic layer was washed with brine and 5% aq. solution of LiCl, filtered and concentrated under reduced pressure to give a dark yellow oil. Column chromatography [SiO_2, EtOAc:MeOH (100:0→80:20)] gave TPBE as the tetrabutylammonium salt as a beige solid (481 mg, 66%). R_f [SiO_2, EtOAc]: 0.1. δ_H (400 MHz, DMSO-d_6): 7.46-7.41 (3H, m, H-2, H-6, Ar-O**H**), 7.29 (2H, d, J=8.3 Hz, H-3, H-5), 7.14 (6H, broad s, H-2'''', H-6''''), 6.95 (6H, t, J=7.4 Hz, H-3'''', H-5''''), 6.83 (3H, t, J=7.2 Hz, H-4''''), 3.57-3.39 (6H, m, 2 × CH_2NCO, CH_2CO), 3.20-3.12 (8H, m, 4 × CH_2N), 2.85-2.75 (4H, m, 2 × CH_2NSO_2), 2.70-2.64 (2H, m, H_2-4'''), 2.01 (3H, s, CH_3Ar), 2.00 (3H, s, CH_3Ar), 1.96-1.90 (1H, m, H-3'''), 1.90 (3H, s, CH_3Ar), 1.80-1.73 (1H, m, H-3'''), 1.63-1.52 (8H, m, 4 × CH_2), 1.36-1.25 (8H, m, 4 × CH_2), 1.23 (3H, s, CH_3-2'''), 0.94 (12H, t, J=7.3 Hz, 4 × CH_3). δ_C (126 MHz, DMSO-d_6): 168.10 (C), 161.92 (q, J(C-^{11}B) = 50.0 Hz, BPh_3), 145.31 (C), 143.99 (C), 135.70 (CH), 135.32 (CH), 127.59 (C), 125.55 (CH), 124.39 (CH), 122.72 (C), 121.91 (CH), 120.93 (C), 120.32 (C), 116.62 (C), 73.93 (C), 57.47 (CH_2), 46.17 (CH_2), 45.91 (CH_2), 40.93 (CH_2), 40.18 (CH_2), 39.97 (CH_2), 31.14 (CH_2), 23.96 (CH_3), 23.02 (CH_2), 20.09 (CH_2), 19.18 (CH_2), 13.46 (CH_3), 12.75 (CH_3), 11.81 (CH_3), 11.77 (CH_3). δ_B (128 MHz, DMSO-d_6): -6.63. LRMS (ESI$^-$): 714 [M^- (borate anion, ^{11}B and ^{13}C), 45%], 713 [M^- (borate anion, ^{11}B and ^{10}B plus ^{13}C), 100], 712 [M^- (borate anion, ^{10}B), 21]. HRMS: 712.3233. $C_{43}H_{46}N_2O_5S^{10}B$ requires [M^- (borate anion)], 712.3262.

Sodium(4-{4'-[2''-(6'''-hydroxy-2''',5''',7''',8'''-tetramethylchroman-2''-yl)acetyl] piperazin-1'-ylsulfonyl}phenyl)triphenylborate, TPBE (sodium salt)

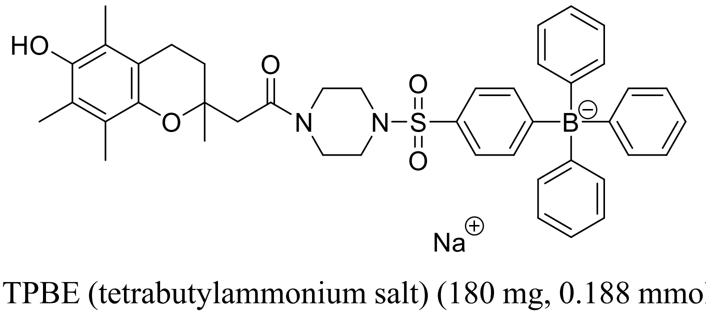

TPBE (tetrabutylammonium salt) (180 mg, 0.188 mmol) was washed through an ion exchange column loaded with sodium cations in MeOH:H_2O (4:1) solution. Concentration of the eluent under reduced pressure yielded TPBE (sodium salt) as a solid (129 mg, 93%). Mp: 206 °C (decompose). ν_{max} (ATR): 3589 (OH), 2926 (C-H), 1618 (C=O). δ_H (400 MHz, DMSO-d_6): 7.45-7.40 (3H, m, H-2, H-6, Ar-OH), 7.28 (2H, d, J=8.3 Hz, H-3, H-5), 7.13 (6H, broad s, H-

2'''', H-6''''), 6.94 (6H, t, J=7.4 Hz, H-3'''', H-5''''), 6.82 (3H, t, J=7.9 Hz, H-4''''), 3.58-3.35 (6H, m, 2 × CH_2NCO, CH_2CO), 2.85-2.73 (4H, m, 2 × CH_2NSO_2), 2.70-2.64 (2H, m, H_2-4'''), 2.00 (3H, s, CH_3Ar), 1.99 (3H, s, CH_3Ar), 1.94-1.90 (1H, m, H-3'''), 1.89 (3H, s, CH_3Ar), 1.80-1.73 (1H, m, H-3'''), 1.22 (3H, s, CH_3-2'''). δ_C (126 MHz, DMSO-d_6): 168.10 (C), 161.91 (q, J (C-^{11}B) = 49.1 Hz, BPh_3), 145.31 (C), 143.99 (C), 135.70 (CH), 135.31 (CH), 127.60 (C), 125.57 (CH), 124.41 (CH), 122.72 (C), 121.92 (CH), 120.93 (C), 120.32 (C), 116.62 (C), 73.93 (C), 46.17 (CH_2), 45.91 (CH_2), 45.06 (CH_2), 40.93 (CH_2), 39.97 (CH_2), 31.14 (CH_2), 23.97 (CH_3), 20.09 (CH_2), 13.46 (CH_3), 12.75 (CH_3), 11.81 (CH_3). δ_B (128 MHz, DMSO-d_6): -6.62. LRMS (ESI$^-$): 714 [M^- (borate anion, ^{11}B and ^{13}C), 42%], 713 [M^- (borate anion, ^{11}B and ^{10}B plus ^{13}C), 100], 712 [M^- (borate anion, ^{10}B), 18]. HRMS: 712.3229. $C_{43}H_{46}N_2O_5S^{10}B$ requires [M^- (borate anion)], 712.3262.

Sodium [4-(morpholin-1'-ylsulfonyl)phenyl]triphenylborate, TPBM (sodium salt).

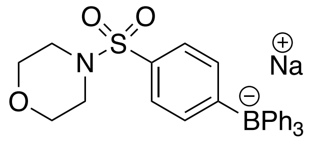

PhMgCl (3.0 mL, 6 mmol, 4 eq.) was added slowly dropwise to a stirred cooled solution (0 °C) of potassium [4-(morpholin-1'-ylsulfonyl)phenyl]trifluoroborate (0.5 g, 1.5 mmol, 1 eq.) in dry THF (20 mL) under argon and stirred for 30 min. The reaction mixture was allowed to warm up to RT and stir for 24 h. The mixture was added slowly into a solution of aqueous solution of Na_2CO_3 (3.23 g in 43 mL of water). The mixture was stirred vigorously for 60 min at RT. The reaction mixture was extracted with EtOAc several times and organic layer was washed with brine. The organic layer was dried (Na_2SO_4) and concentrated under reduced pressure to give as a crude. Column chromatography [SiO_2, Petroleum ether:EtOAc (1:3 to 1:9), EtOAc (100%), MeOH:EtOAc (5:95 to 20:80)] to give the corresponding sodium salt as a solid (254 mg, 36%). δ_H (500 MHz, DMSO-d_6): 7.45 (2H, broad d, J=7.5 Hz, H-2 and H-6), 7.31 (2H, d, J=8.0 Hz, H-3 and H-5), 7.17 – 7.13 (6H, broad s, H-2', H-6'), 6.97 (6H, t, J=7.5 Hz, H-3', H-5'), 6.83 (3H, t, J=7.5 Hz, H-4'), 3.61 (4H, t, J=4.5 Hz, 2 × CH_2), 2.97 (4H, t, J=4.5 Hz, 2 × CH_2). δ_C (126 MHz, DMSO-d_6): 162.02 (q, J (C-^{11}B) = 48.7 Hz, BPh_3), 135.65, 135.32, 127.20, 128.58, 124.54, 121.94, 65.24, 46.00. LRMS (ESI$^-$): 469 [M^-(borate anion, ^{11}B and ^{13}C), 32%], 468 [M^-(borate anion, ^{11}B and ^{10}B plus ^{13}C), 100%], 467 [M^-(borate anion, ^{10}B), 20%]. HRMS (ESI$^-$): 467.1831. $C_{28}H_{27}NO_3S^{10}B$ requires [M^-(borate anion)], 467.1847.

Sodium(4-{4'-[3"-(4"',4"'-Difluoro-1"',3"',5"',7"'-tetramethyl-4"'-bora-3"'a,4"'a-diaza-*s*-indacene-8"'-yl)propanoyl]piperazin-1'-ylsulfonyl}phenyl)triphenylborate, TPBBODIPY (sodium salt).

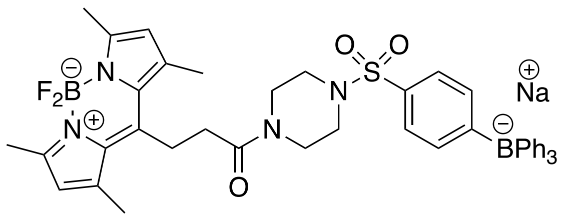

EDCI (31.0 mg, 0.164 mmol, 2.0 eq) was added to a solution of 3-(4,4-Difluoro-1,3,5,7-tetramethyl-4-bora-3a,4a-diaza-*s*-indacene-8-yl)propionic acid [7] (39.0 mg, 0.123 mmol, 1.5 eq), amine **5** (58.0 mg, 0.082 mmol, 1.0 eq) and DMAP (1.0 mg, 0.008 mmol, 0.1 eq) in dry CH_2Cl_2 (3 mL). The solution was stirred at RT for 4 h under an atmosphere of argon. After this time the solution diluted with CH_2Cl_2 (10mL) and washed with 1 M hydrochloric acid (2 × 30 mL), dried over sodium sulfate and concentrated under vacuum. The residue was purified by column chromatography using a 12 g Agela cartridge eluting CH_2Cl_2:MeOH (100:0) increasing to CH_2Cl_2:MeOH (93:7). The product was then ion exchanged to the sodium salt and the product purified again by column chromatography using a 12 g Agela cartridge eluting CH_2Cl_2:MeOH (100:0) increasing to CH_2Cl_2:MeOH (90:10) to give the product as a red solid (13.0 mg, 20%). δ_H (400 MHz: MeCN-d_3): 7.55-7.51 (2H, m, H-3 + H-5), 7.36 (2H, broad d, J = 7.8 Hz, H-2 + H-6), 7.25-7.23 (6H, m, Ar-H), 7.03 (6H, broad t, J = 7.1 Hz, Ar-H), 6.90 (3H, broad t, J = 7.1 Hz, Ar-H), 6.13 (2H, s, CH), 3.60 (2H, t, J = 5.2 Hz, CH_2NCO), 3.44 (2H, t, J = 4.9 Hz, CH_2NCO), 3.25-3.20 (2H, m, CH_2), 2.90-2.84 (4H, m, 2 × CH_2NSO_2), 2.59 (2H, t, J = 8.3 Hz, CH_2), 2.44 (6H, s, 2 × Me), 2.33 (6H, s, 2 × Me). δ_C (100 MHz: MeCN-d_3): 170.10 (C), 163.30 (q, J = 49.6 Hz, C), 154.94 (C), 147.15 (C), 142.44 (C), 136.9 (CH), 136.57 (CH), 132.12 (C), 129.15 (C), 126.79 (q, J = 2.4 Hz, CH), 125.66 (q, J = 2.4 Hz, CH), 123.18 (CH), 122.64 (CH), 47.16 (CH_2), 46.96 (CH_2), 45.28 (CH_2), 41.81 (CH_2), 34.33 (CH_2), 24.46 (CH_2), 16.51 (CH_3), 14.55 (t, J = 2.3 Hz, CH_3). δ_B(128 MHz: MeCN-d_3): 0.48 (t, J = 35.2 Hz, BF_2), -6.54 (s). HRMS (ESI$^-$): 769.3342. $C_{44}H_{45}{}^{11}B_2F_2N_4O_3S$ requires [M^-(borate anion)], 769.3372.

Sodium (4-{4'-[7"-(diethylamino)coumarin-3"-carbonyl]piperazin-1'-ylsulfonyl} phenyl) triphenylborate, TPBCoumarin (sodium salt).

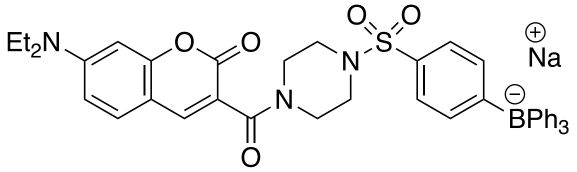

EDCI (27.0 mg, 0.14 mmol, 2.0 eq) was added to a solution of 7-(diethylamino)coumarin-3-carboxylic acid (27.0 mg, 0.10 mmol, 1.5 eq), amine **5** (50.0 mg, 0.070 mmol, 1.0 eq) and DMAP (1.0 mg, 0.008 mmol, 0.1 eq) in dry CH_2Cl_2 (3 mL). The solution was stirred at R.T. for 4 h under an atmosphere of argon. After this time the solution diluted with CH_2Cl_2 (10mL) and washed with 1 M hydrochloric acid (2 × 30mL), dried over sodium sulfate and concentrated under vacuum. The residue was purified by column chromatography using a 12 g Agela cartridge eluting CH_2Cl_2:MeOH (100:0) increasing to CH_2Cl_2:MeOH (85:15). The product was then ion exchanged to the sodium salt and the product purified again by column chromatography using a 12 g Agela cartridge eluting CH_2Cl_2:MeOH (100:0) increasing to CH_2Cl_2:MeOH (85:15) to give the product as a red solid (14.0 mg, 27%). δ_H (400 MHz: MeCN-d$_3$): 7.72 (1H, s, H-4), 7.53 (2H, broad s, Ar-H), 7.38-7.35 (3H, m, Ar-H + H-8), 7.24 (6H, broad s, Ar-H), 7.25-7.23 (6H, m, Ar-H), 7.04 (6H, broad t, J = 7.1 Hz, Ar-H), 6.89 (3H, broad t, J = 7.1 Hz, Ar-H), 6.70 (1H, d, J = 8.9 Hz, H- 6), 6.53 (1H, s, H-5), 3.68 (2H, broad s, CH_2NCO), 3.46 (2H, broad s, CH_2NCO), 3.45 (4H, q, J = 6.6 Hz, NCH_2), 2.94 (4H, broad s, 2 × CH_2NSO_2), 1.17 (6H, t, J = 6.6 Hz, , NCH_2CH_3). δ_C (100 MHz: MeCN-d$_3$): 165.96 (C), 163.35 (q, J = 49.6 Hz, C), 160.17 (C), 158.18 (C), 152.84 (C), 145.11 (CH), 137.00 (CH), 136.62 (CH), 131.04 (CH), 129.38 (C), 126.86 (q, J = 2.4 Hz, CH), 125.72 (q, J = 2.4 Hz, CH), 123.23 (CH), 116.63 (C), 110.50 (CH), 108.41 (C), 97.50 (CH), 47.07 (Broad CH_2), 45.50 (CH_2), 42.09 (Broad CH_2), 12.69 (CH_3). δ_B (128 MHz: MeCN-d$_3$): -6.55 (s). HRMS (ESI$^-$): 710.2849. $C_{42}H_{41}{}^{11}BN_3O_5S$ requires [M$^-$(borate anion)], 710.2865.

2. Black Lipid Membrane Experiments

Planar Black Lipid Membranes (BLM) were formed from a 10 mg/ml solution of 1,2-dioleoyl-*sn*-glycero-3-phosphocholine (DOPC) (Avanti Polar Lipids) in n-decane (Aldrich). The solution was painted across the 200 μm aperture of a Delrin cup (Warner Instruments, Hamden, CT). Both *cis* (voltage command side) and *trans* (virtual ground) compartments of the cup contained 150 mM NaCl, 2 mM $CaCl_2$, 10 mM Tris-HCl pH 7.4. Stock solutions (10 mM) of

each compound were prepared in methanol and prior to experiment compounds were diluted 1 – 10 fold in water and added to both compartments of the cuvette to give the indicated final concentrations. All measurements were performed at room temperature.

Charge accumulated BLM, and currents flowing across BLM were recorded with a Planar Lipid Bilayer Workstation (Warner Instruments). The *cis* compartment was connected to the head stage input and the trans compartment was held at virtual ground via a pair of matched Ag/AgCl electrodes. Signals from voltage-clamped BLM were low-pass-filtered at 2. 1 Hz and in some experiments at 2.1 kHz using an eight-pole Bessel filter LPF-8 (Warner Instruments) and recorded after digitization through a homemade analog-to-digital converter assembled using Data Translation Board. Data were acquired using homemade acquisition software (Elena Pavlov) and analyzed using Origin software.

Total charge, accumulated on lipid bilayer, was estimated by integrating area under the signal trace using a threshold of 10 % of the peak current. Accumulation of the charge, induced by the influence of the tested compound, was determined by subtraction of the total charge under control conditions from the total charge, estimated in the presence of the tested compound. Selectivity of the current, induced by the lipophilic anions, was measured by estimating the zero current potential (reversal potential (E_{rev})) under the 10:1 (*cis*/*trans*) concentration gradient of the tested compound.

3. Submitochondrial Particle Experiments

Bovine heart mitochondria were prepared from *Bos taurus* heart tissue as described previously [11] and stored as ~5 g pellets at -20˚C. To prepare submitochondrial particles (SMPs), a pellet of bovine heart mitochondria was thawed overnight at 4˚C, resuspended in 10 mM Tris-SO_4, 250 mM sucrose at pH 7.0 (final volume 120 ml, pH corrected at 20 ˚C, buffer A), and refrozen. Then 40 ml of the suspension was thawed at 4˚C, centrifuged (11,300 × g, 12 min, 4˚C), the dark red supernatant discarded, and the pellet was resuspended in buffer A to 40 ml. The pH was adjusted to 9 on ice by the dropwise addition of 2.5 M Tris, and the sample was incubated on ice for 15 min then recentrifuged (37,900 × g, 12 min, 4˚C). The supernatant was discarded, and the pellet was twice resuspended in buffer A to 40 ml and recentrifuged (11,300 × g, 12 min, 4˚C). Then 10 mM $MgSO_4$, was added and the sample was sonicated on ice (Sonicator 3000, Misonix, 19 mm probe, ten 15 s bursts with 1 min intervals, 150 W). The sonicated material was centrifuged (27,100 × g, 20 min, 4˚C), and the pellet discarded. Then, 1 mM NADH was added, and the sample was incubated for 1 h on ice. The SMPs were collected by centrifugation (82,000 × g, 30 min, 4˚C) then twice resuspended to 4 ml in buffer A and

recentrifuged. Finally, the SMPs were resuspended to ~7 mg protein/ml (determined using the bicinchoninic acid (BCA) assay) and stored as aliquots at -20˚C. The yield of SMPs was typically ~30 mg protein.

To prepare the hexadodecylpyridinium (HDP) tetraphenylborate (TPB) ion pair, sodium tetraphenylborate NaTPB (Aldrich, 10 mM, 171.1 mg) in 50 ml MilliQ water and hexadodecylpyridinium chloride (HDPCl) (Fluka, 10 mM, 179.0 mg) in 50 ml MilliQ water were mixed and cooled on dry ice for ~30 min [12]. The precipitate was gravity filtered through Whatman paper, washed with distilled water and dried at room temperature giving HDP-TPB (75 mg) as a white solid. To prepare the ion-selective electrode (ISE) membrane, polyvinylchloride (PVC) (Aldrich, 320 mg) and dioctyl phthalate (DOP, Aldrich, 270 mg) were dissolved in tetrahydrofuran (THF; 10 mL). This was mixed with HDP-TPB (75 mg) dissolved in THF (3 mL) and poured into a 60 cm^2 glass Petri dish and the THF evaporated at room temperature overnight, leaving a clear membrane ~ 0.2 mm thick. Disks ~4 mm in diameter were prepared using a scalpel and glued to the polished end of 3 – 4 cm lengths of PVC electrical sleeving (Farnell, cat no. PVC-4-CL) with THF. The electrode was then filled with NaTPB (10 mM) and soaked overnight in the same solution. A Pt wire soldered to a coaxial cable into the PVC sleeve and this electrode and a Ag/AgCl reference electrode were inserted into a stirred 2 ml incubation chamber, thermostatted at 37˚C. The output from the electrodes were monitored using a PowerLab Data acquisition system via a front-end pH amplifier and analysed using Chart software, all from ADInstruments. To measure the uptake of tetraphenylborate compounds by submitochondrial particles (SMPs), SMPs (0.2 mg protein/ml) were incubated at 37˚C in a stirred and thermostatted chamber in 1 ml ST buffer (250 mM Sucrose, 10 mM Tris-SO4, pH 7.4). The electrode was calibrated by five sequential additions of 1 µM of the compound to be assessed and NADH (1 mM), and carbonylcyanide-*p*-trifluoromethoxyphenylhydrazone (FCCP) (1 µM) were added where indicated.

4. Cell Culture Experiments

C2C12 cells (mouse myoblast cell line; European Collection of Animal Cell Cultures), HeLa cells (Human cervix epitheloid carcinoma cell line; European Collection of Animal Cell Cultures), and Cos7 cells (COS-7-InVitrus cell line; European Collection of Animal Cell Cultures) were cultured in Dulbecco's modified Eagle's medium (DMEM; Invitrogen) supplemented with 10% (v/v) fetal calf serum, 100 U/mL penicillin and 100 μg/mL streptomycin at 37 °C in a humidified 95% air and 5% CO_2 environment. To assess the effect of TPBE on cell viability we used the CellTiter 96® AQ_{ueous} Non-Radioactive Cell

Proliferation Assay (Promega). Cells were seeded in a 96 well plate (10000 cells in 200 μL medium per well), allowed to grow for 24 h when the medium was aspirated and tested compounds were added in fresh medium (200 μL per well). After 24 h the medium was aspirated, the plate was washed with medium then medium (100 μL), and MTS solution (20 μL) were added to each well. Cells were incubated at 37°C in a humidified 95% air and 5% CO_2 environment for 2 h. After that time the absorbance was read at 490 nm. This showed that TPBE did not show toxicity at concentrations of 10 µM or below (Fig S2), indicating concentration ranges that can be used safely.

5. Confocal Microscopy

For incubations with TPBCoumarin, C2C12, Hela and Cos7 (50,000) cells were seeded in 27 mm glass bottomed dishes (Thermo #150682) in complete DMEM (high glucose + 10% dFBS + 1% PenStrep) and grown overnight. Next day, cells were washed twice with 1 ml complete FluoroBrite™ DMEM (Thermo A1896701) and in some cases incubated with 20 µM pitstop2 (Abcam) for 30 minutes. After this incubation, cells were placed in a Zeiss LSM880 confocal system equipped with a Zeiss Plan-Achromat 63x/1.4NA oil immersion objective. 3D images were acquired after 10 min of incubation with 100 nM of TPBCoumarin using a 405 nm laser and corresponding filters (Coumarin 343 setup). For the localisation studies, cells were incubated with 500 nM of both MitoTracker Deep Red FM (Invitrogen) and Lysotracker Red DND-99 (Invitrogen) for 5 min prior to imaging.

For analysis of the uptake of TPBBODIPY the cells were prepared as described above and then placed in 5% CO_2 at 37˚C imaging incubator for acquisition with a Dragonfly Spinning Disk imaging system (Andor Technologies Ltd.) composed by a Nikon Ti-E microscope, Nikon 100x TIRF ApoPlan and an Ixon EMCCD camera. 3D images were acquired every 5 sec using a 488 nm or 568 nm laser and corresponding filters, after a few images the compounds were added at 100 nM. Images were analysed and videos mounted at 7 fps rate using the Imaris v.9.1.2 software (Bitplane AG, Zurich, Switzerland).

For video analysis of the uptake of TPBCoumarin and TPBBodipy C2C12 cells were prepared the cells were prepared as described above and placed in a Zeiss LSM880 confocal system equipped with a Zeiss Plan-Achromat 100x/1.4NA oil immersion objective. Images were acquired every 10 sec for 20 min using a 405 nm laser and corresponding filters (Coumarin 343 setup) for TPBCoumarin, and 488 nm laser and corresponding filters for TPBBodipy. TPBCoumarin or TPBBodipy (both 100 nM) were added after a few images were taken. Videos

were mounted at 5 fps rate using the Imaris v.9.1.2. software (Bitplane AG, Zurich, Switzerland).

6. Author contribution

Kishore K. Gaddale Devanna, Justyna M. Gawel and Stuart T. Caldwell carried out the synthetic chemistry.

Tracy A. Prime, Filip Cvetko, Cristiane Benincá and Andrew Harrison carried out the cell incubations and the confocal microscopy.

Kishore K. Gaddale Devanna, Justyna M. Gawel and Tracy Prime carried out the biochemical incubations with SMPs and cells.

Evgeny V. Pavlov and Alexander Negoda carried out the black lipid membrane experiments.

Andrew M. James and Michael P. Murphy supervised the biological experiments

Richard C. Hartley designed structures and supervised the synthetic chemistry

7. References

1. Franzke, A.; Pfaltz, A., Synthesis of Functionalized Borate Building Blocks for the Anionic Derivatization of Neutral Compounds. *Synthesis* **2008,** *2*, 245-252.
2. Franzke, A.; Pfaltz, A., Zwitterionic Iridium Complexes with P,N-Ligands as Catalysts for the Asymmetric Hydrogenation of Alkenes. *Chemistry Europe* **2011,** *17*, 4131-4144.
3. Ishiyama, T.; Murata, M.; Miyaura, N., Palladium(0)-Catalyzed Cross-Coupling Reaction of Alkoxydiboron with Haloarenes: A Direct Procedure for Arylboronic Esters. *J. Org. Chem. 60*, 7508-7510.
4. Lennox, A. J. J.; Lloyd-Jones, G. C., Preparation of Organotrifluoroborate Salts: Precipitation-Driven Equilibrium under Non-Etching Conditions. *Angewandte Chemie (International ed* **2012,** *51*, 9385-9388.
5. Bagutski, V.; Ros, A.; Aggarwal, V. K., Improved method for the conversion of pinacolboronic esters into trifluoroboratesalts: facile synthesis of chiral secondary and tertiary trifluoroborates. *Tetrahedron* **2009,** *65*, 9956-9960.
6. Scott, J. W.; Bizzarro, F. T.; Parrish, D. R.; Saucy, G., Syntheses of (2R,4'R,8'R)-alpha-tocopherol and (2R,3'E,7'E)-alpha-tocotrienol. *Helv Chim Acta* **1976,** *59* (1), 290-306.
7. Thivierge, C.; Han, J.; Jenkins, R. M.; Burgess, K., Fluorescent proton sensors based on energy transfer. *The Journal of organic chemistry* **2011,** *76* (13), 5219-28.
8. Tateishi, H.; Tsuji, A. B.; Kato, K.; Sudo, H.; Sugyo, A.; Hanakawa, T.; Zhang, M. R.; Saga, T.; Arano, Y.; Higashi, T., Synthesis and evaluation of (11)C-labeled coumarin analog as an imaging probe for detecting monocarboxylate transporters expression. *Bioorganic & medicinal chemistry letters* **2017,** *27* (21), 4893-4897.
9. Wrackmeyer, B., Carbon-13 NMR Spectroscopy of Boron Compounds. *Progress in NMR Spectroscopy* **1979,** *12*, 227-259.
10. Noronha, G.; Barrett, K.; Cao, J.; Gritzen, C.; Gong; Hood, J.; Mak, C. C.; McPherson, A.; Pathak, V. P.; Renick, J.; Soll, R.; Splittgerber, U.; Wrasidlo, W.; Zeng, B.; Zhao, N.; Dneprovskaia, E. Benzotriazine inhibitors of kinases. 2005.
11. Walker, J. E.; Skehel, J. M.; Buchanan, S. K., Structural analysis of NADH: ubiquinone oxidoreductase from bovine heart mitochondria. *Methods in enzymology* **1995,** *260*, 14-34.
12. Shoukry, A. F.; Badawy, S. S.; Issa, Y. M., Performance Characteristics and Regeneration of a Tetraphenylboron(III) Selective Electrode. *Analytical chemistry* **1987,** *59*, 1078-1081.

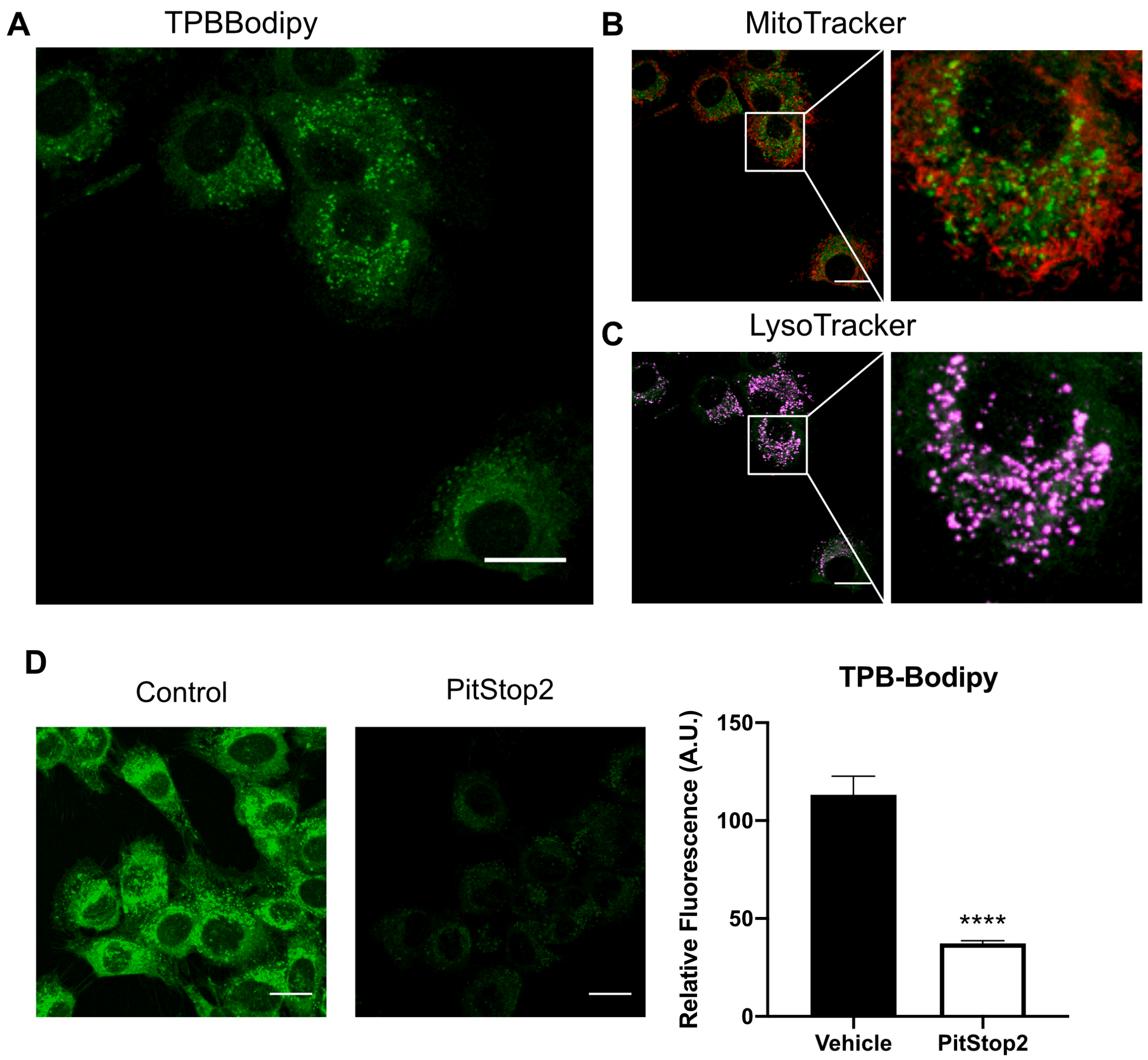

Figure S1 Cell distribution of TPBBODIPY.

Cell distribution of TPBBodipy. (A) C2C12 cells were incubated with 100 nM TPBBodipy (green). Distribution imaged 10 min after addition. (B) Distribution of MitoTracker (red) compared with TPBBodipy (green). (C) Distribution of LysoTracker (magenta) compared with TPBBodipy (green). Colocalization is shown in white. (D) Cells were incubated with 20 μM Pitstop2 for 30 min before addition of TPBBodipy and compared with control. The bar chart shows mean ± SEM of 3 independent experiments. **** $p < 0.0001$ by Student's t-test. Scale bar = 20 μm.

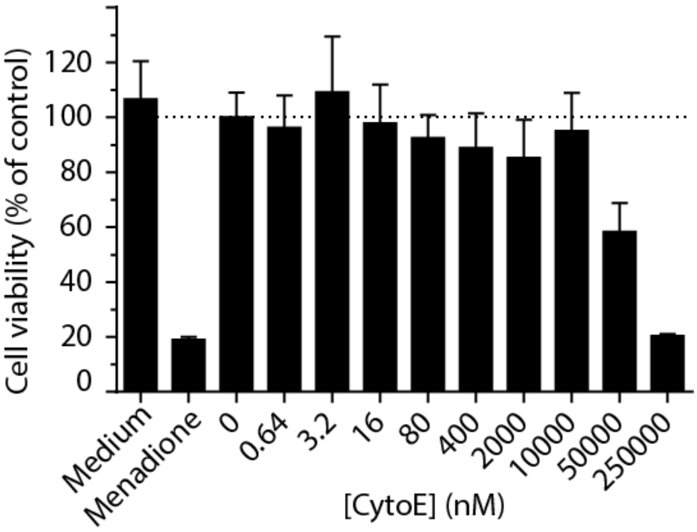

Figure S2 Cell toxicity of TPBE.

The toxicity to cells of TPBE was assessed by incubating C2C12 cells for 24 h with various concentrations of TPBE. Then cell viability was assessed as described in the materials and methods. Data are expressed as a percentage of a control incubation where cells were treated with the same level of ethanol carrier as used to dissolve TPBE. Cells without any additions of TPBE or of ethanol are also shown. Menadione (50 μM) was used to induce cell death as a positive control. Data are means ± SD n = 4.

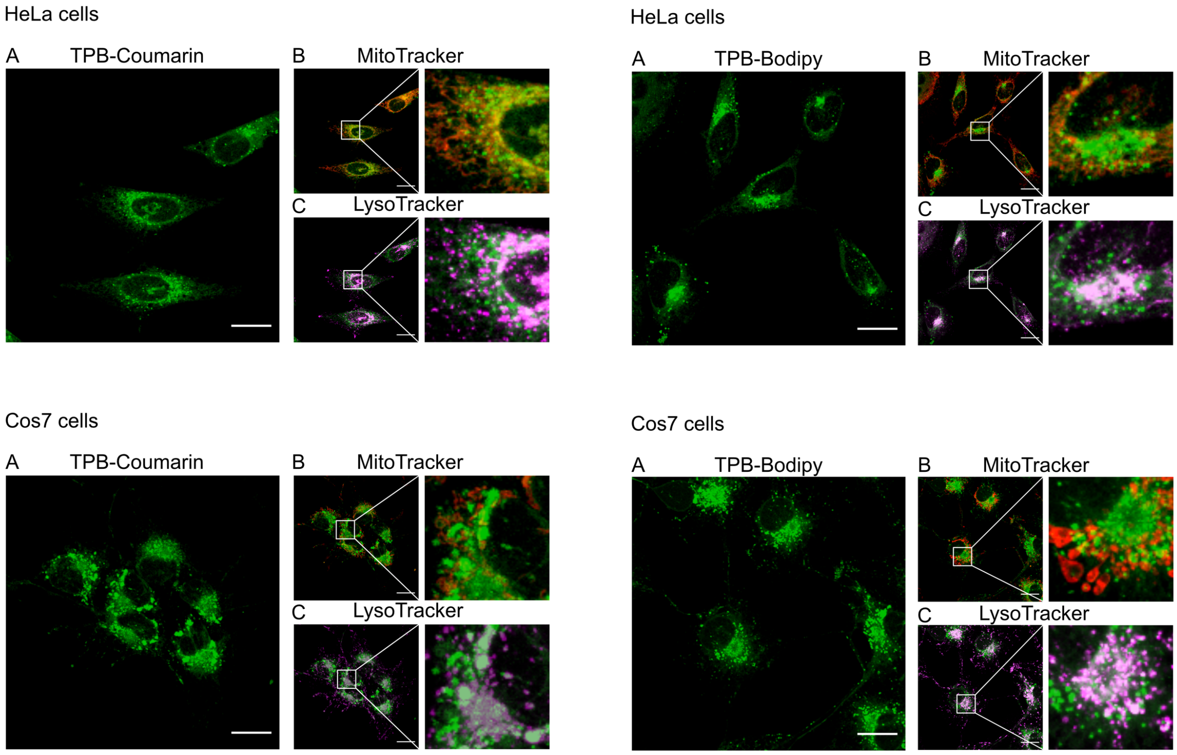

Figure S3 Cell distribution of TPBCoumarin and TPBBODIPY in HeLa and Cos7 cells

Cell distribution of TPBCoumarin. (Top left) Hela cells and (Bottom left) Cos7 cells (A) Cells were incubated with 100 nM TPBCoumarin (green). Distribution imaged 10 min after addition. (B) Distribution of MitoTracker (red) compared with TPBCoumarin (green). (C) Distribution of LysoTracker (magenta) compared with TPBCoumarin (green).

Cell distribution of TPBBODIPY. (Top right) Hela cells and (Bottom right) Cos7 cells (A) Cells were incubated with 100 nM TPBBodipy (green). Distribution imaged 10 min after addition. (B) Distribution of MitoTracker (red) compared with TPBBodipy (green). (C) Distribution of LysoTracker (magenta) compared with TPBBodipy (green). Colocalization is shown in white. Scale bar = 20 μm.

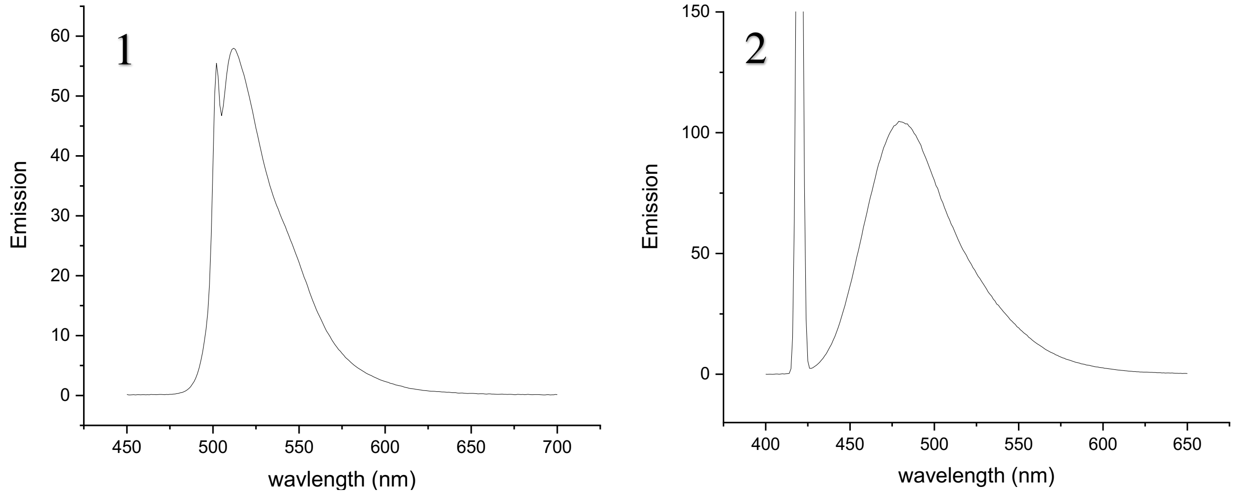

Figure S4 Fluorescence emission of TPBBODIPY and TPBCoumarin

Fluorescence emission of 1) TPBBODIPY (10 μM in pH 7.4 buffer) excitation 500 nm.
2) TPBCoumarin (10 μM in pH 7.4 buffer) excitation 420 nm.

Movie Legends

Movie S1. TPBCoumarin uptake in C2C12 cells. The cells were exposed to 100 nM of TPBcoumarin at the 2-s mark on the video and incubated for a further 15 min. The video is shown at 5 fps and each frame is 10 s apart in real time.

Movie S2. TPBBodipy uptake in C2C12 cells. TPBBodipy uptake in C2C12 cells. The cells were exposed to 100 nM of TPBBodipy after 15 s and incubated for a further 5 min. The video is shown at 7 fps and each frame is 5 s apart in real time.
