# Supplementary material for: Tetra-arylborate lipophilic anions as targeting groups
Source: Chem Commun (Camb). 2021 Feb 18;57(25):3147–50. doi: 10.1039/d0cc07924c (PMC8062962; doi:10.1039/d0cc07924c)

Electronic Supplementary Material (ESI) for Chemical Communications.
This journal is © The Royal Society of Chemistry 2021

Supporting information 2 for

Tetra-Arylborate Lipophilic Anions as Targeting Groups

Kishore Kumar Gaddale Devanna[1], Justyna M. Gawel[1], Tracy A. Prime[2], Filip Cvetko[2], Cristiane Benincá[2], Stuart T. Caldwell[1], Alexander Negoda[3], Andrew Harrison[2], Andrew M. James[2], Evgeny V. Pavlov[4], Michael P. Murphy[2,5,*] and Richard C. Hartley[1,*]

[1]School of Chemistry, University of Glasgow, Glasgow, G12 8TA, UK.

[2]MRC Mitochondrial Biology Unit, Hills Road, Cambridge CB2 0XY, UK.

[3]Department of Physiology and Biophysics, Dalhousie University, Halifax, Nova Scotia, Canada.

[4]New York University, College of Dentistry, Department of Molecular Pathobiology, 345 East 24th Street, New York, NY 10010, USA

[5]Department of Medicine, University of Cambridge, Cambridge, UK.

*Correspondence: Richard.Hartley@glasgow.ac.uk mpm@mrc-mbu.cam.ac.uk

Contents of Supplementary Information 2: NMR Spectra of

Tert-butyl 4-(4’-bromophenylsulfonyl)piperazine-1-carboxylate **1** S2

Methyl 4-(4’-bromophenylsulfonyl)piperazine-1-carboxylate **2** S3

Potassium {4-[4’-(methoxycarbonyl)piperazin-1’-ylsulfonyl]phenyl}trifluoroborate **3** S4

Tetrabutylammonium {4-[4’-(methoxycarbonyl)piperazin-1’- ylsulfonyl]phenyl}triphenylborate **4** S6

Tetrabutylammonium [4-(piperazin-1’-ylsulfonyl)phenyl]triphenylborate **5** S8

TPBE (tetrabutylammonium salt) S10

TPBE (sodium salt) S12

TPBM (sodium salt) S14

TPBBODIPY (sodium salt) S15

TPBCoumarin (sodium salt) S17

Tert-butyl 4-(4'-bromophenylsulfonyl)piperazine-1-carboxylate 1

^{1}H NMR spectrum

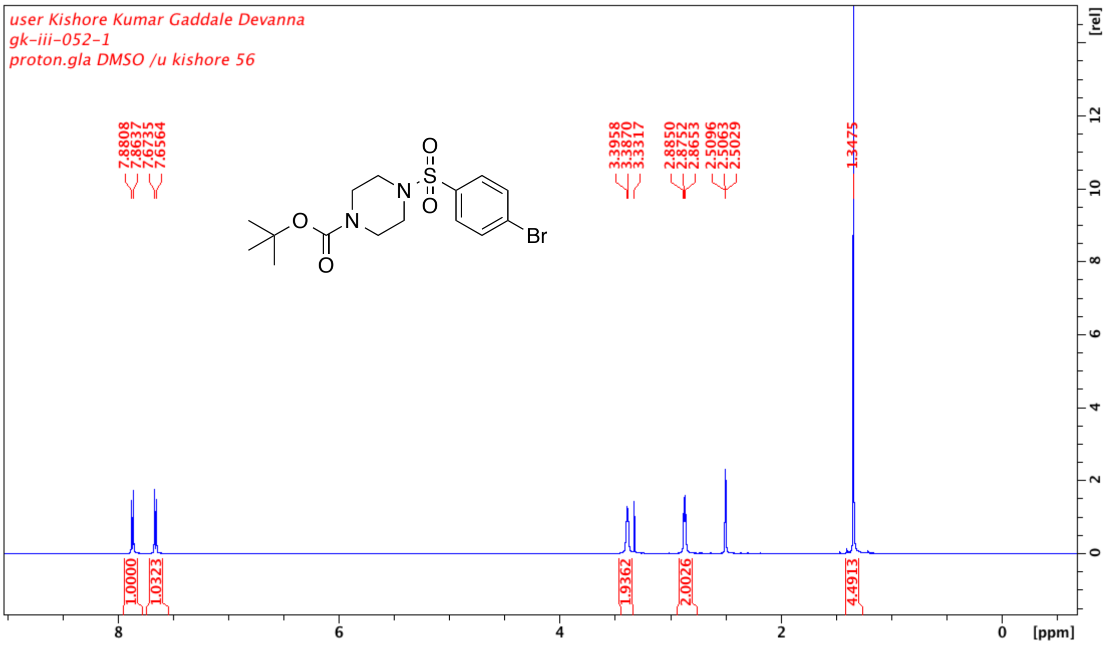

^{13}C NMR spectrum

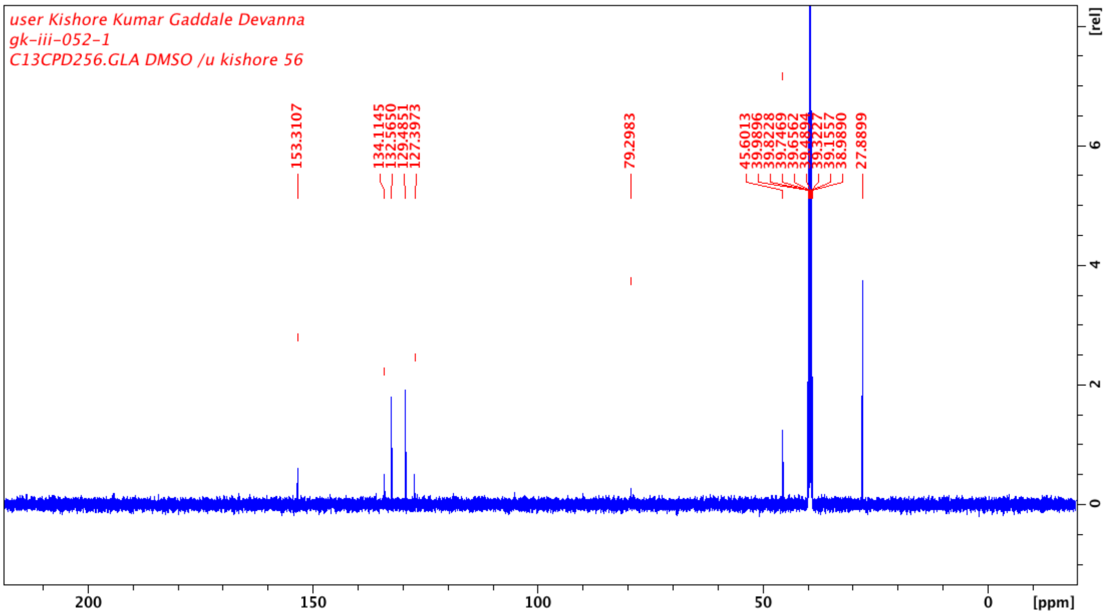

Methyl 4-(4’-bromophenylsulfonyl)piperazine-1-carboxylate 2

^{1}H NMR spectrum

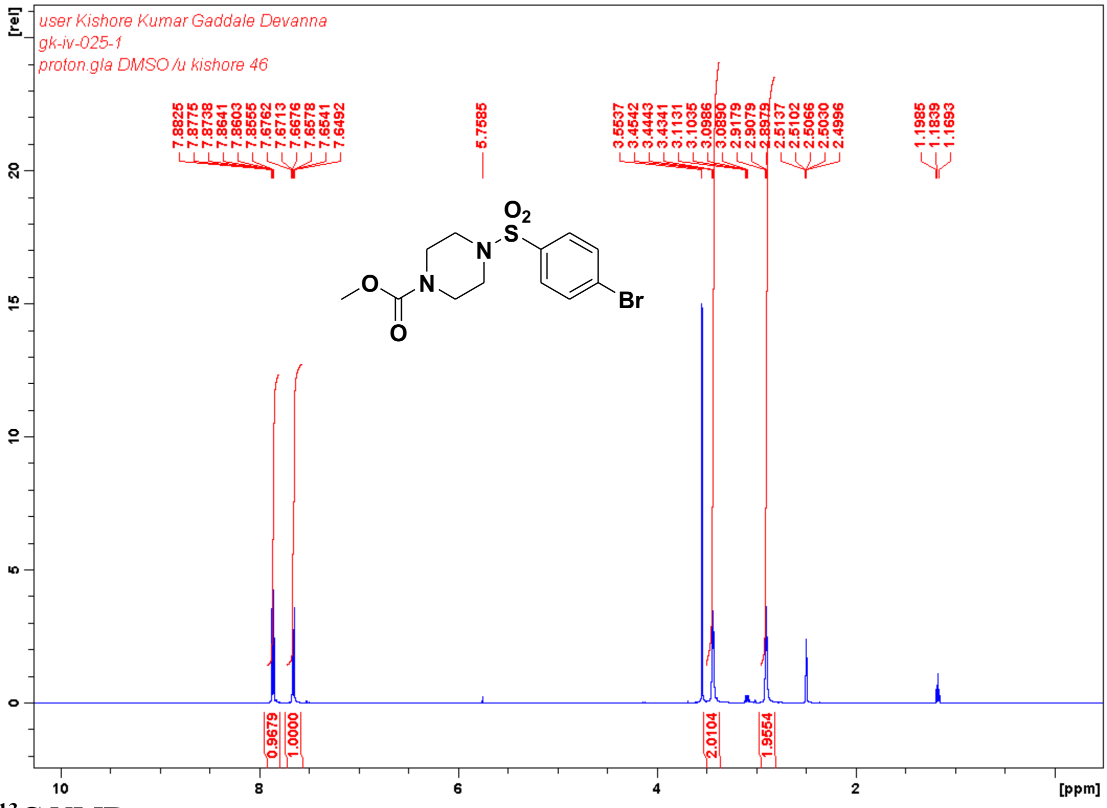

^{13}C NMR spectrum

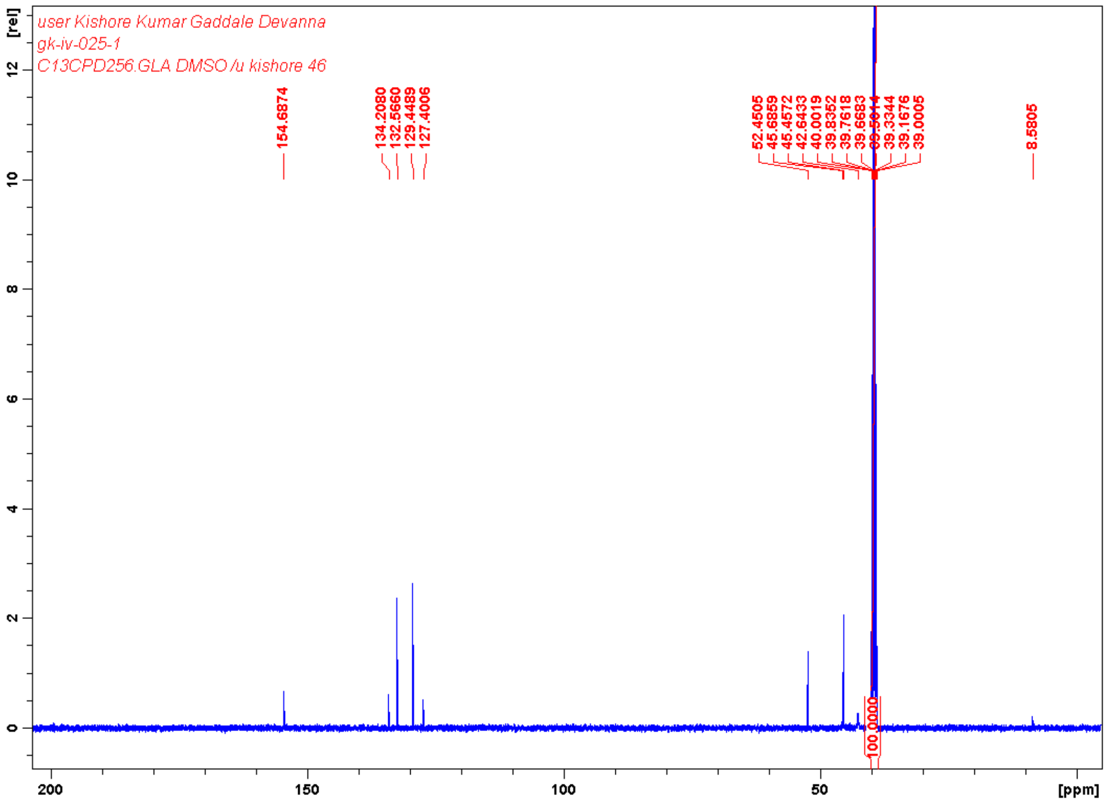

Potassium {4-[4’-(methoxycarbonyl)piperazin-1’-ylsulfonyl]phenyl}trifluoroborate 3

^{1}H NMR spectrum

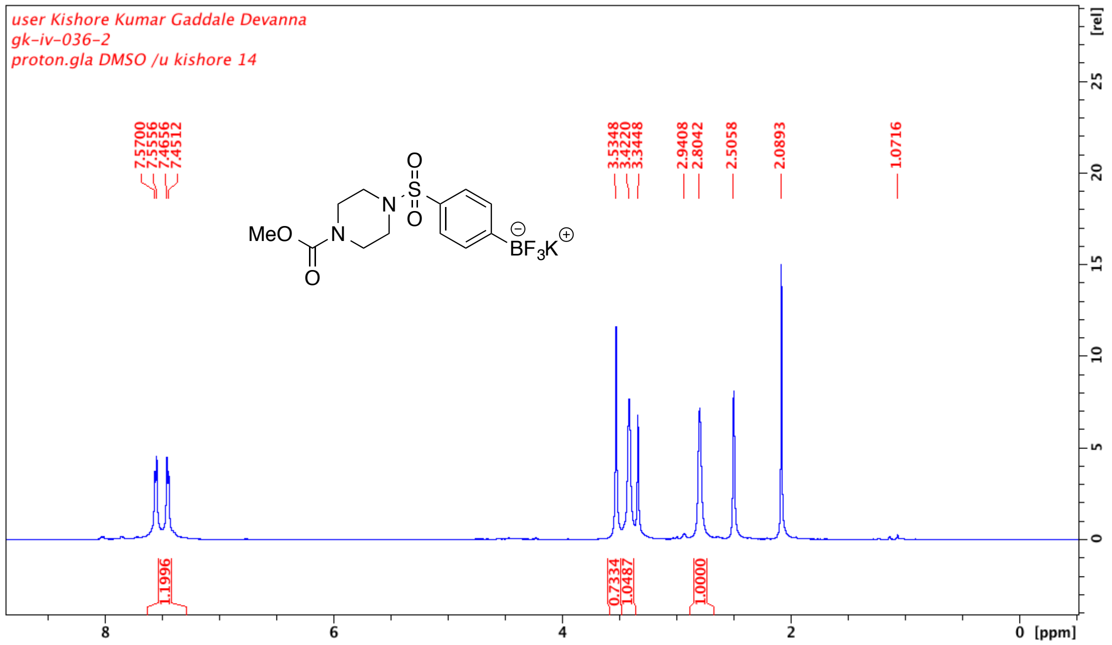

^{13}C NMR spectrum

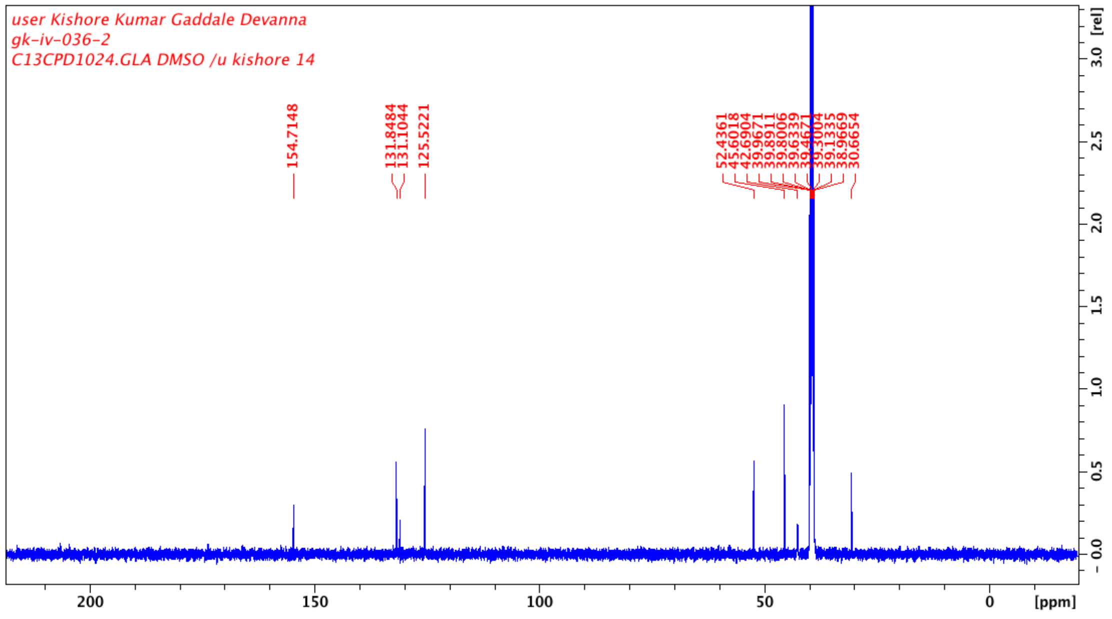

^{19}F NMR spectrum

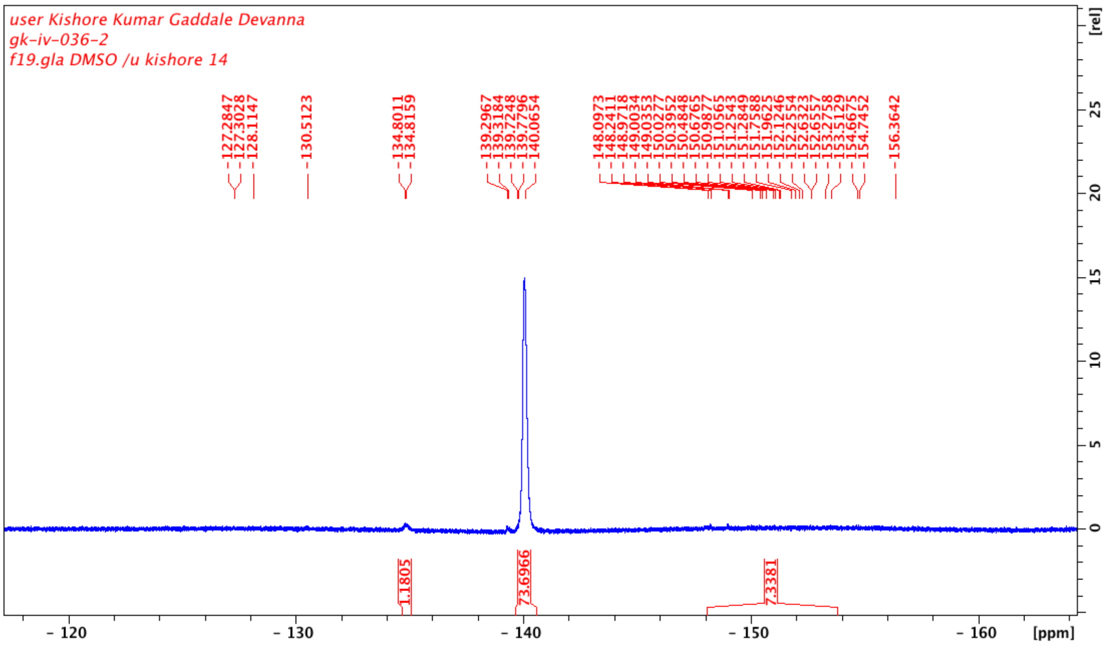

Tetrabutylammonium

{4-[4'-(methoxycarbonyl)piperazin-1'- ylsulfonyl]phenyl}triphenylborate 4

^{1}H NMR spectrum

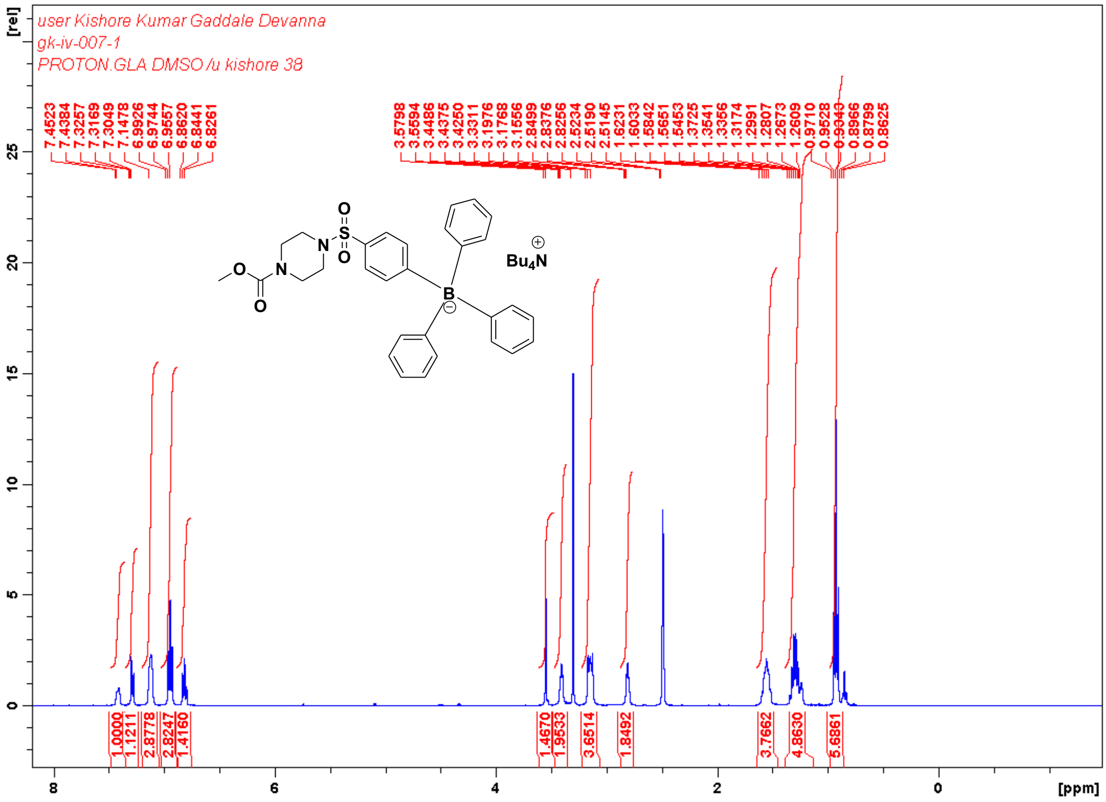

^{13}C NMR spectrum

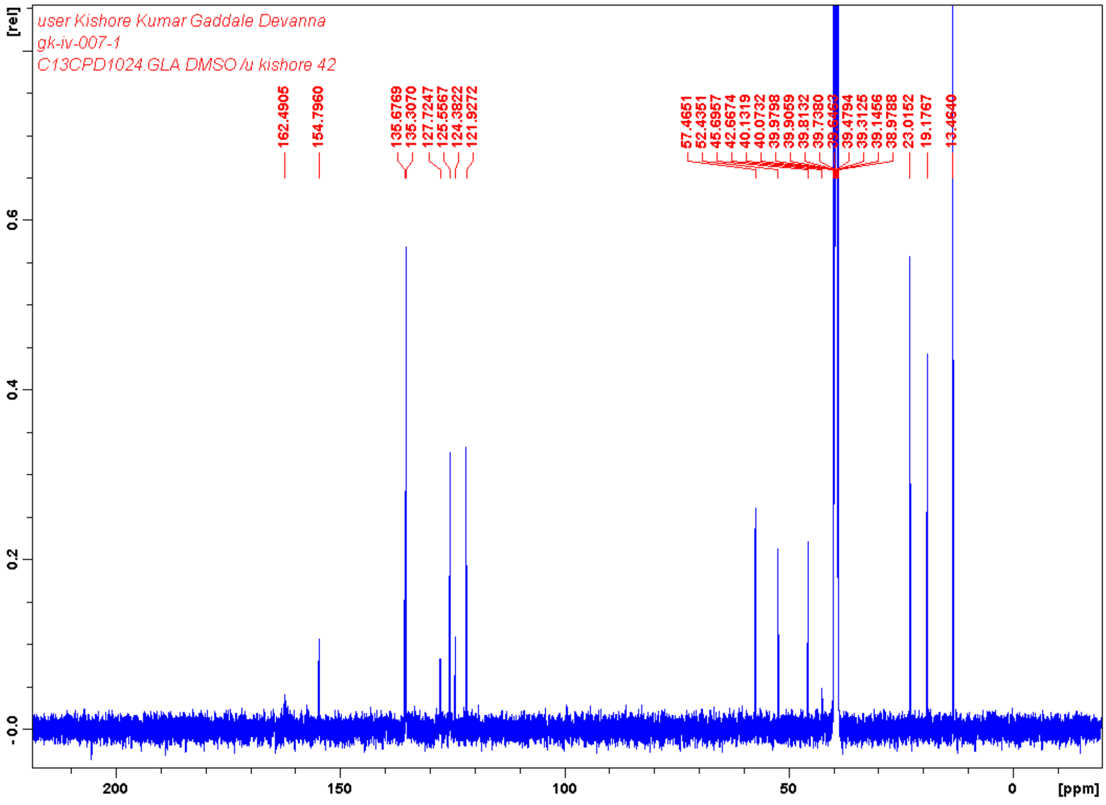

^{11}B NMR spectrum

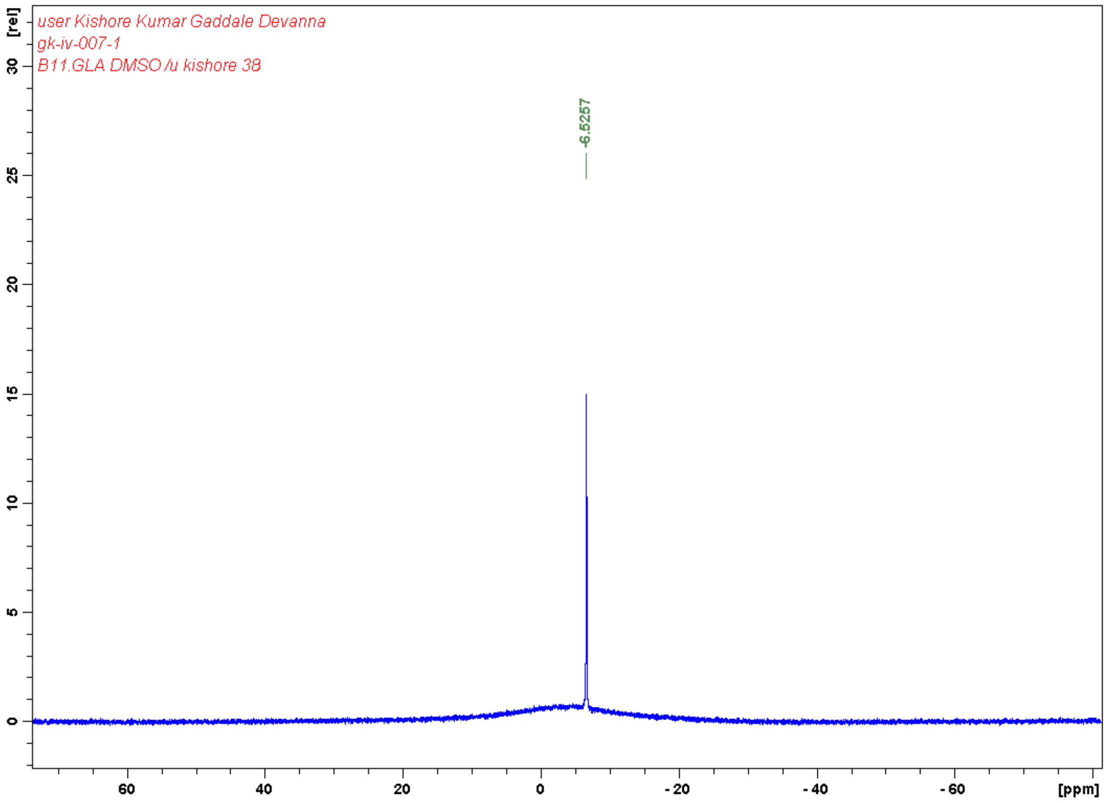

Tetrabutylammonium [4-(piperazin-1’-ylsulfonyl)phenyl]triphenylborate 5

^{1}H NMR spectrum

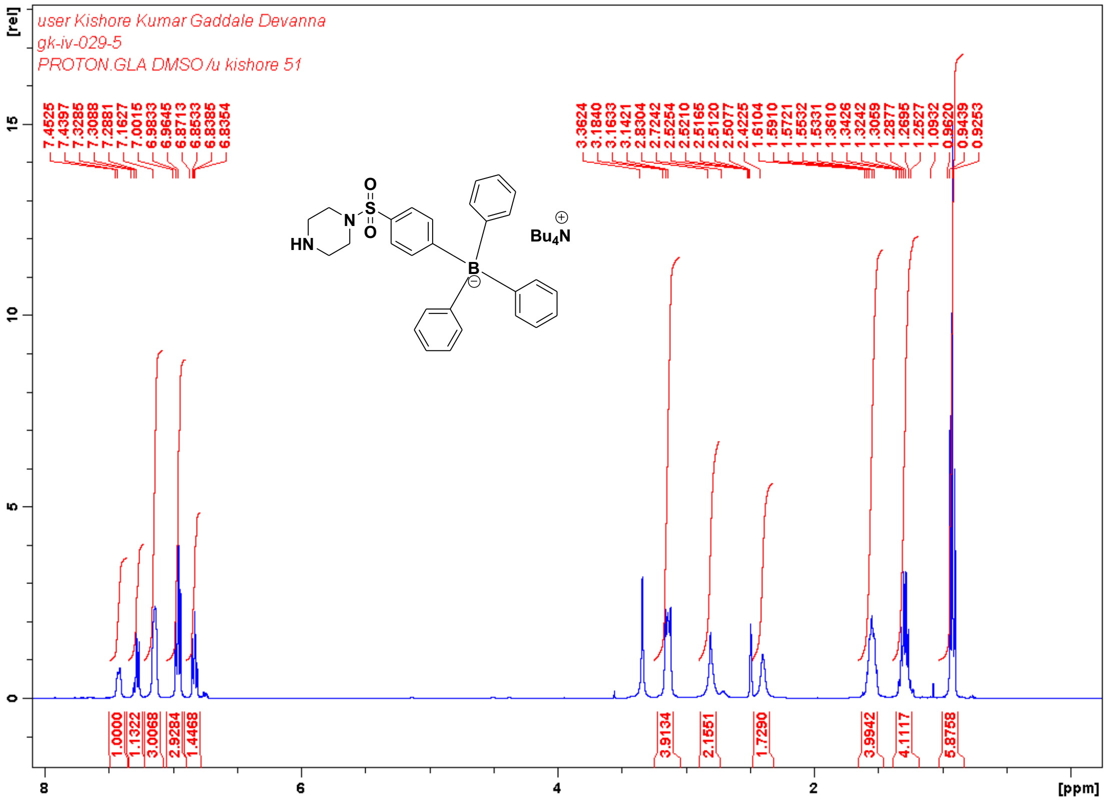

^{13}C NMR spectrum

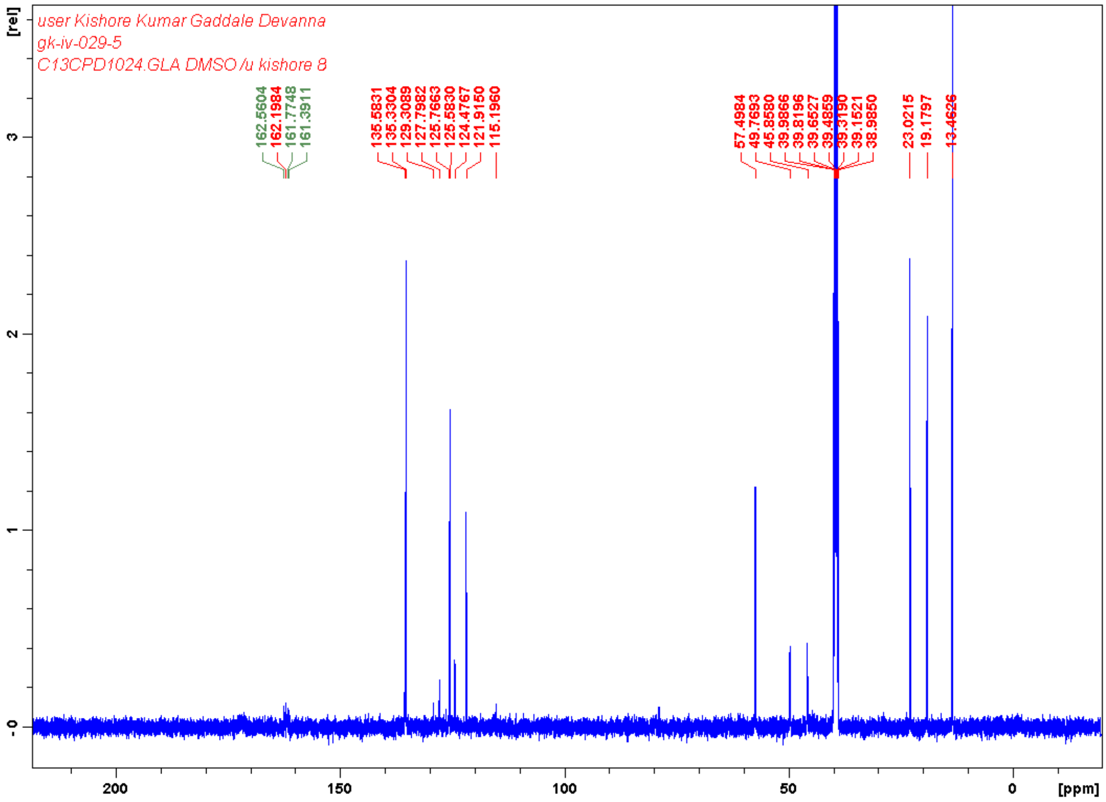

^{11}B NMR spectrum

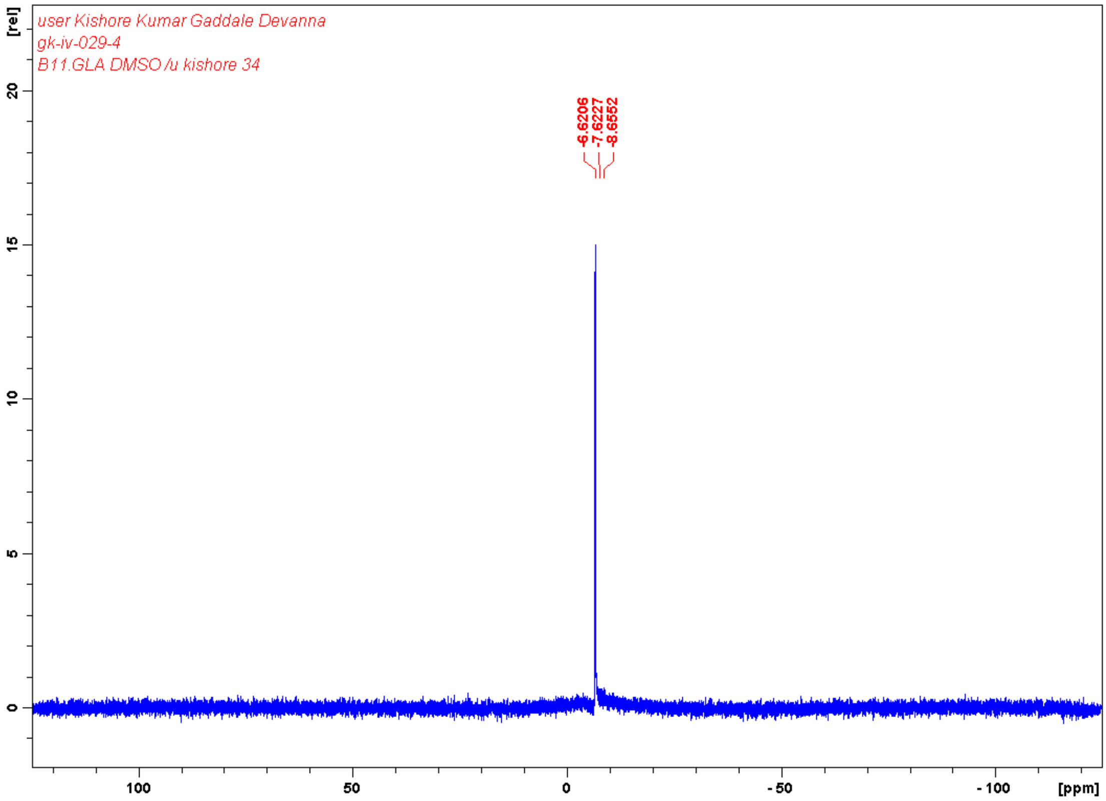

Tetrabutylammonium (4-{4’-[2’’-(6’’’-hydroxy-2’’’,5’’’,7’’’,8’’’-tetramethylchroman-2’’’-yl)acetyl]piperazin-1’-ylsulfonyl}phenyl)triphenylborate, TPBE (tetrabutylammonium salt)

^{1}H NMR spectrum

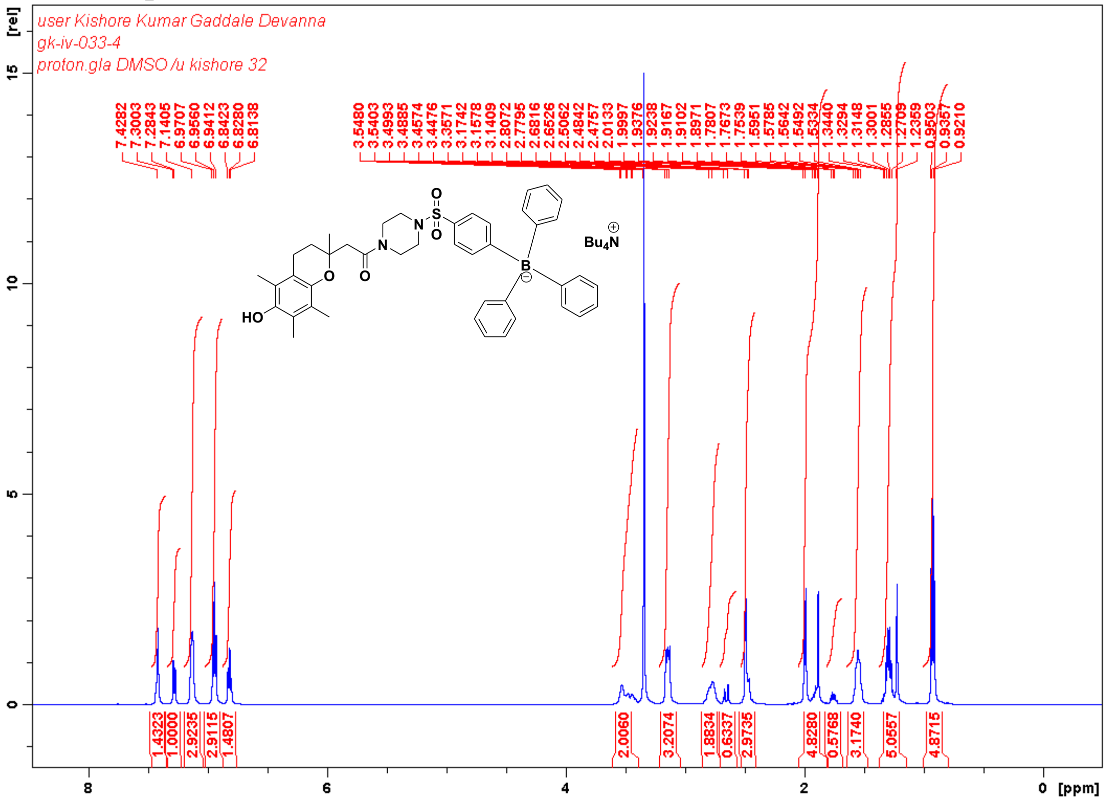

^{13}C NMR spectrum

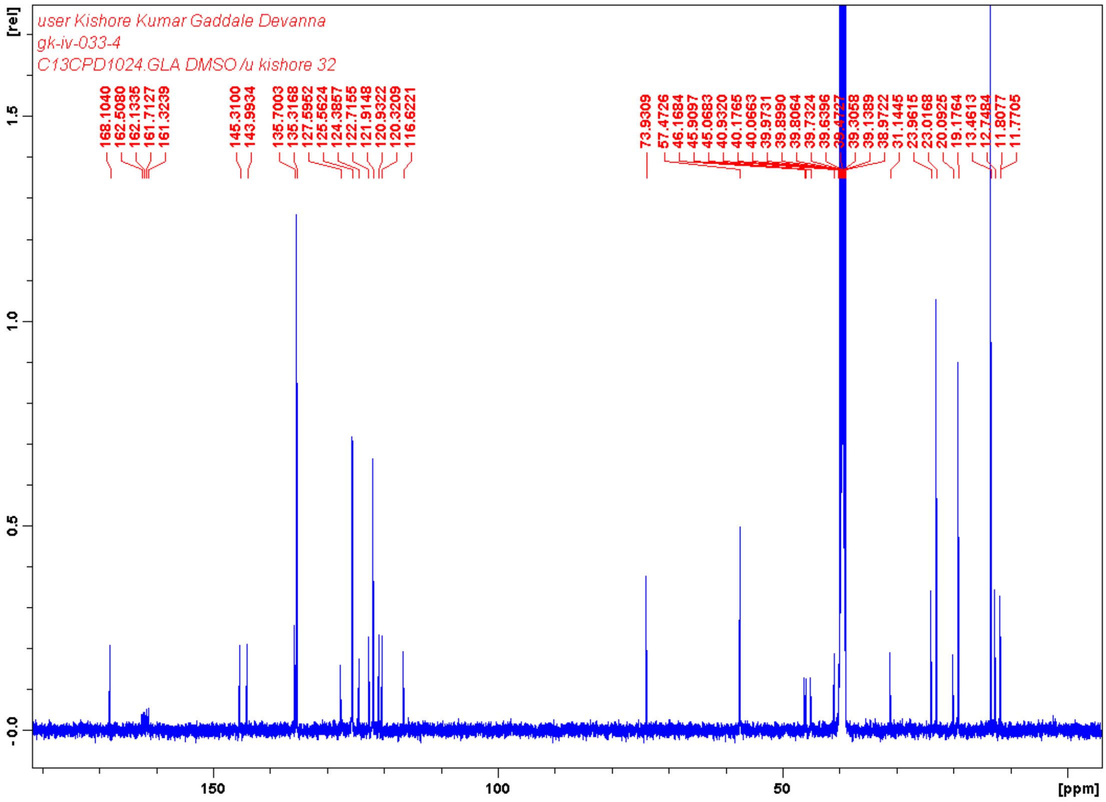

^{11}B NMR spectrum

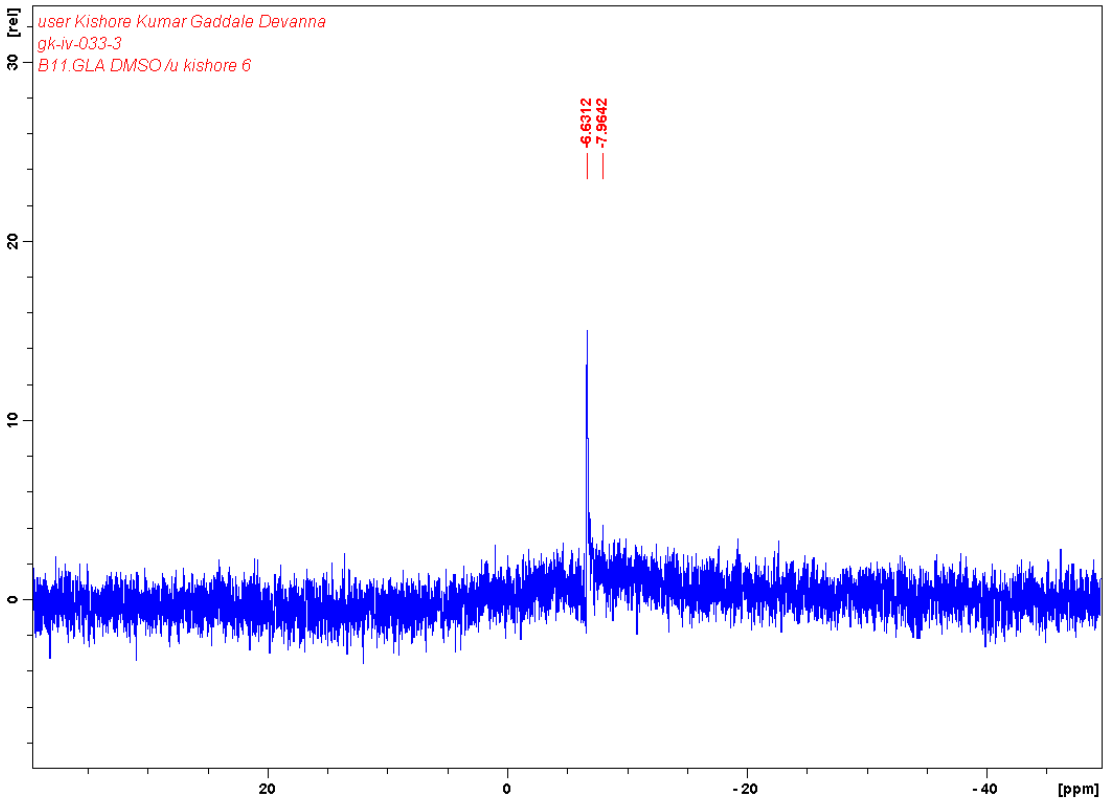

Sodium (4-{4'-[2''-(6'''-hydroxy-2''',5''',7''',8'''-tetramethylchroman-2''-yl)acetyl]piperazin-1'-ylsulfonyl}phenyl)triphenylborate, TPBE (sodium salt)

^{1}H NMR spectrum

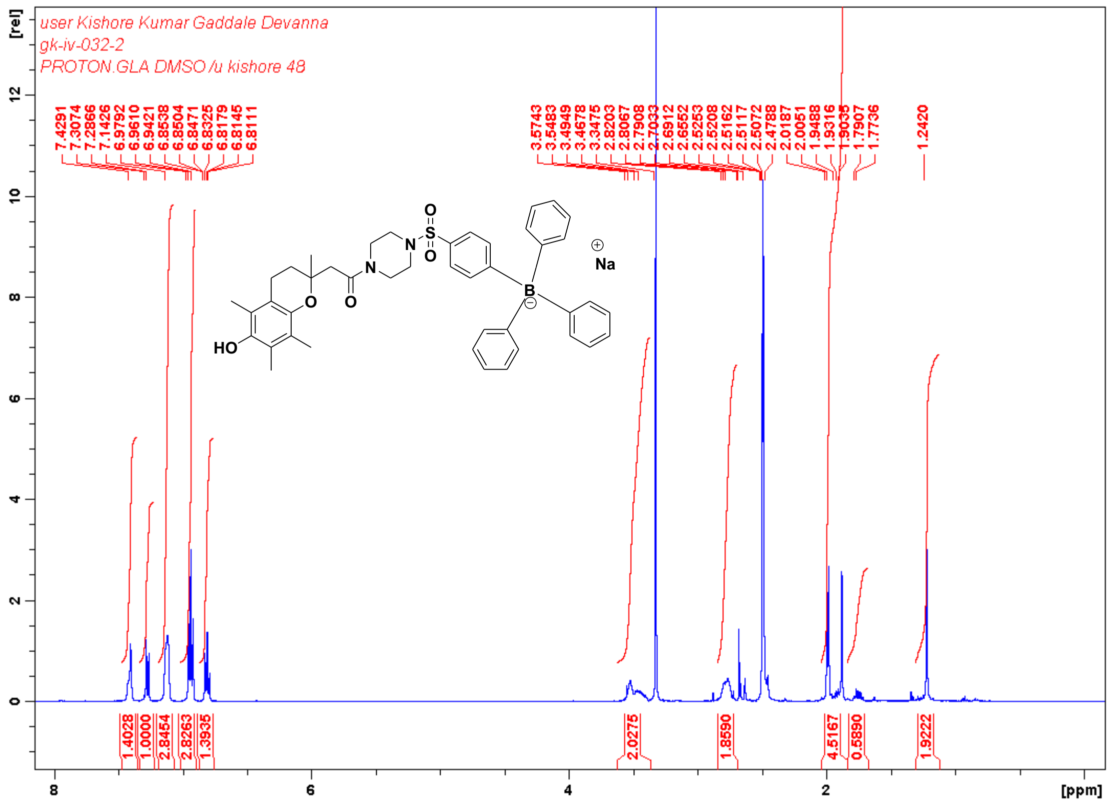

^{13}C NMR spectrum

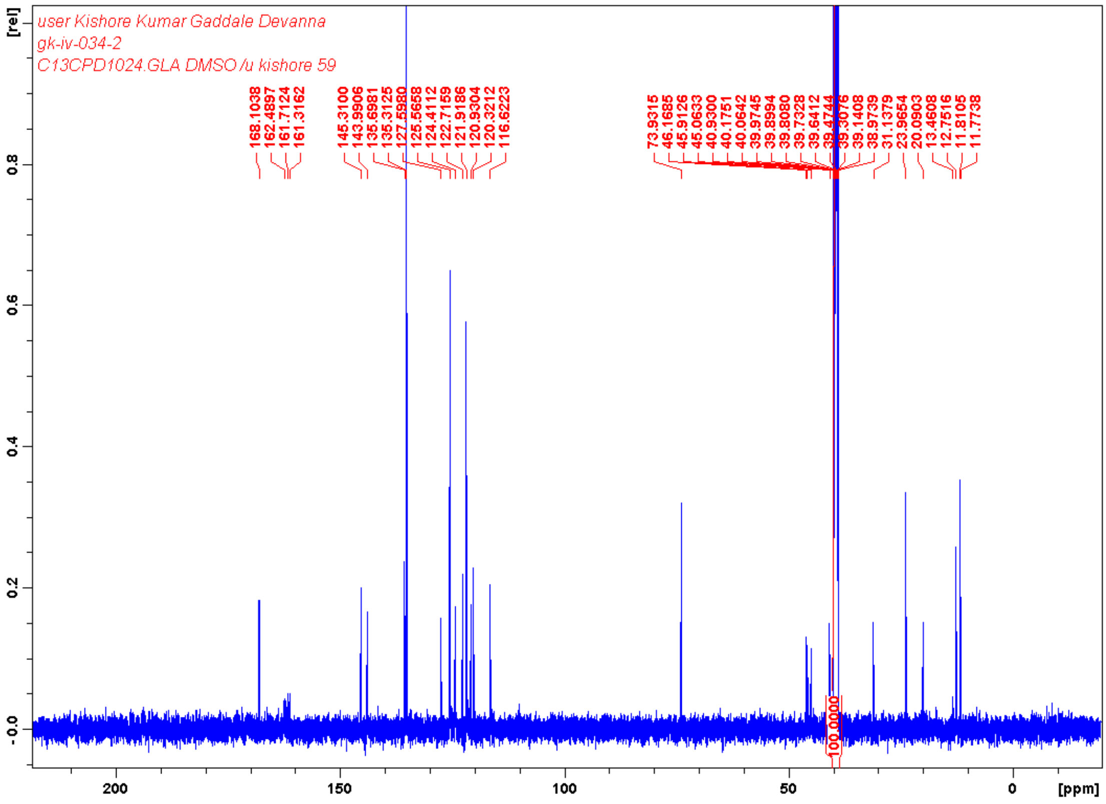

^{11}B NMR spectrum

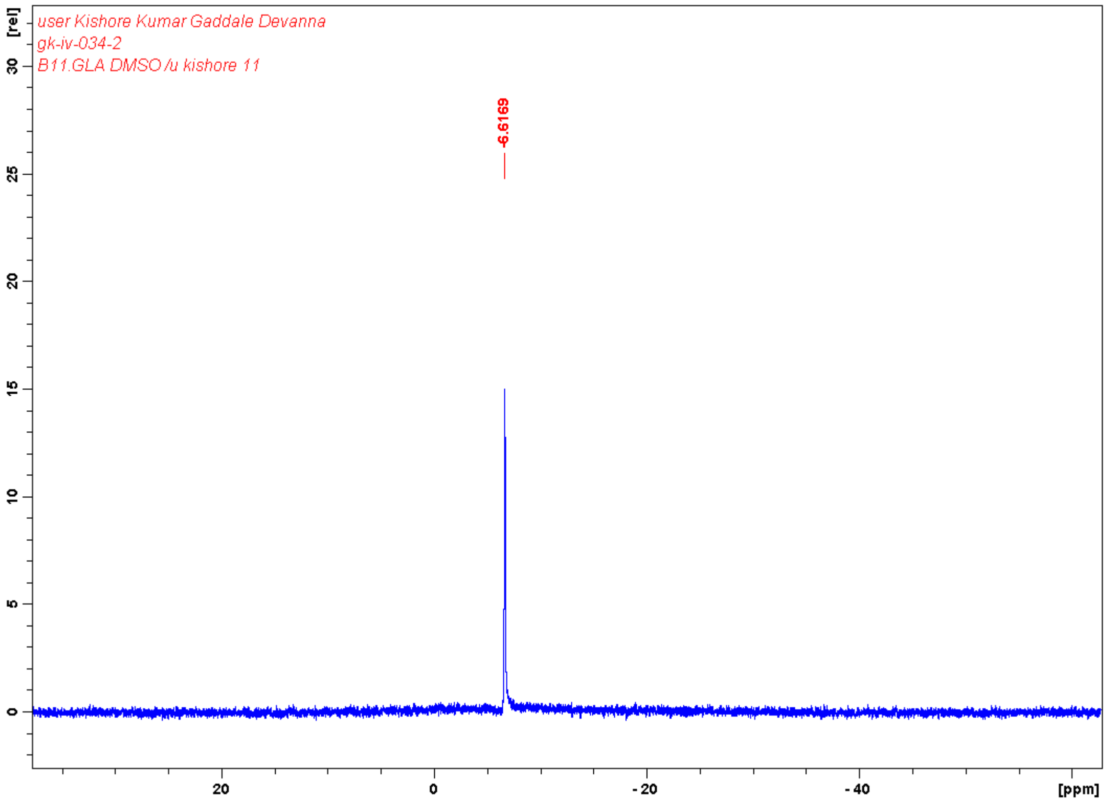

Sodium [4-(morpholin-1’-ylsulfonyl)phenyl]triphenylborate, TPBM (sodium salt).

^{1}H NMR spectrum

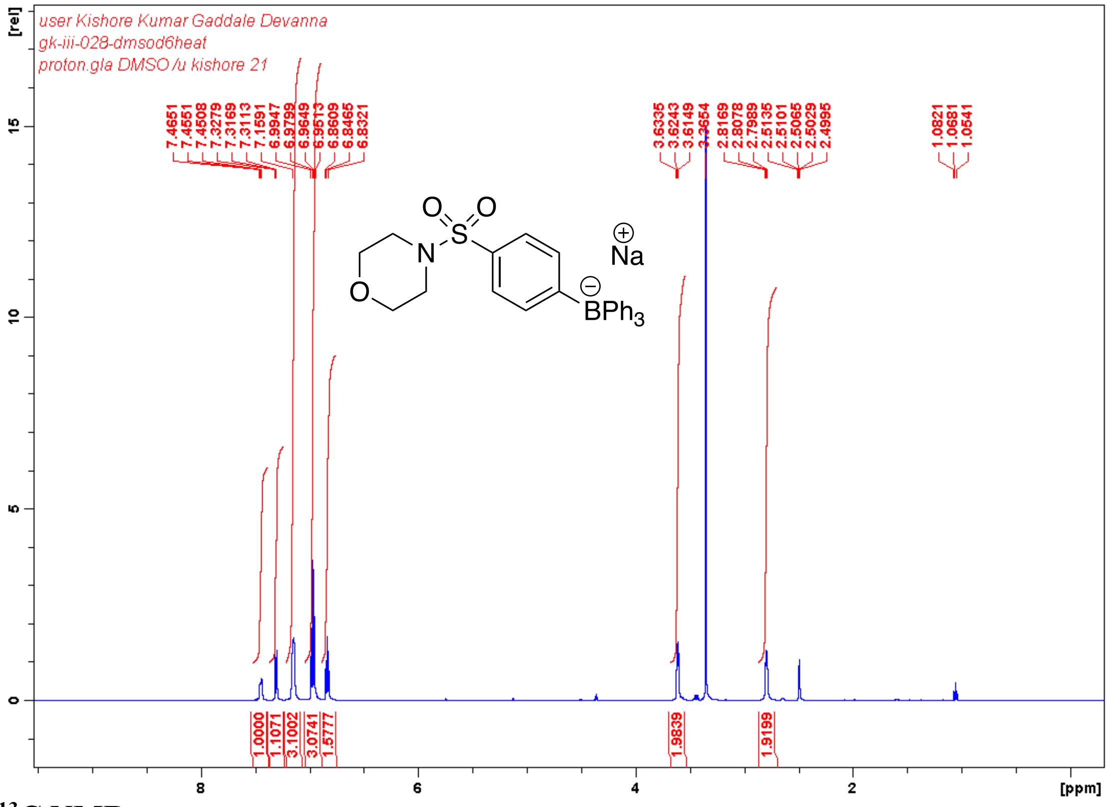

^{13}C NMR spectrum

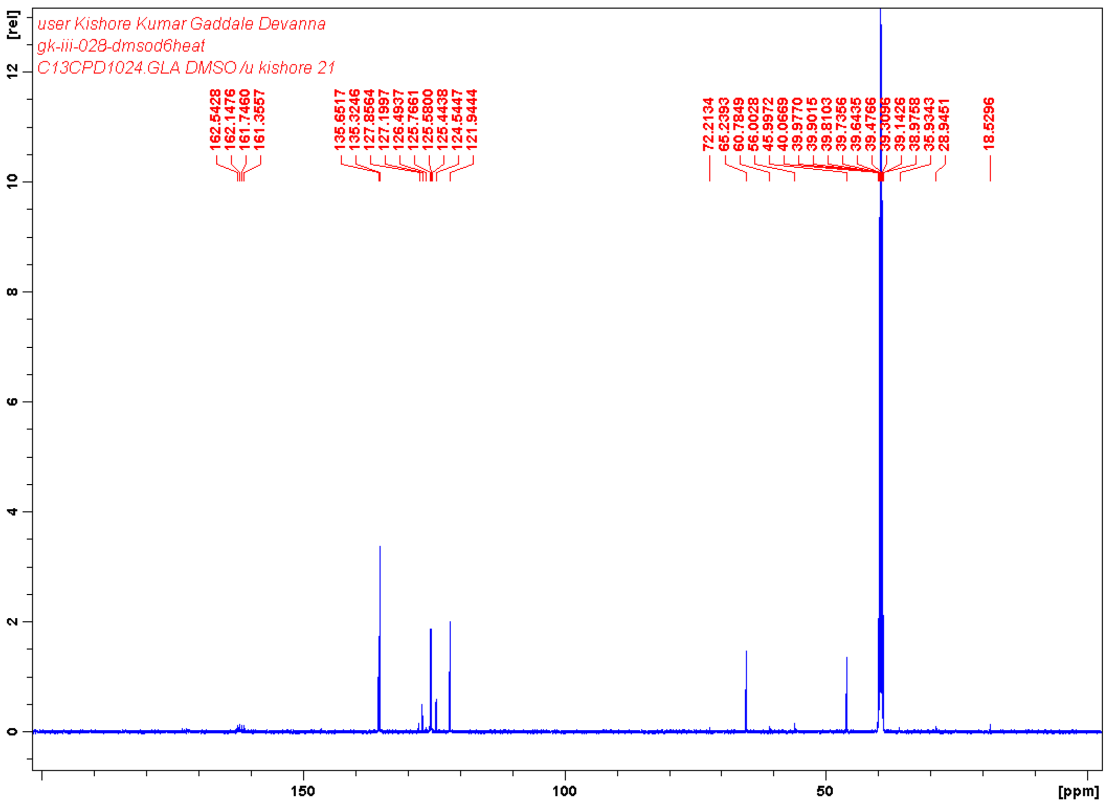

Sodium (4-{4'-[3''-(4''',4'''-Difluoro-1''',3''',5''',7'''-tetramethyl-4'''-bora-3'''a,4'''a-diaza-*s*-indacene-8'''-yl)propanoyl]piperazin-1'-ylsulfonyl}phenyl)triphenylborate, TPBBODIPY (sodium salt). ^{1}**H NMR Spectrum**

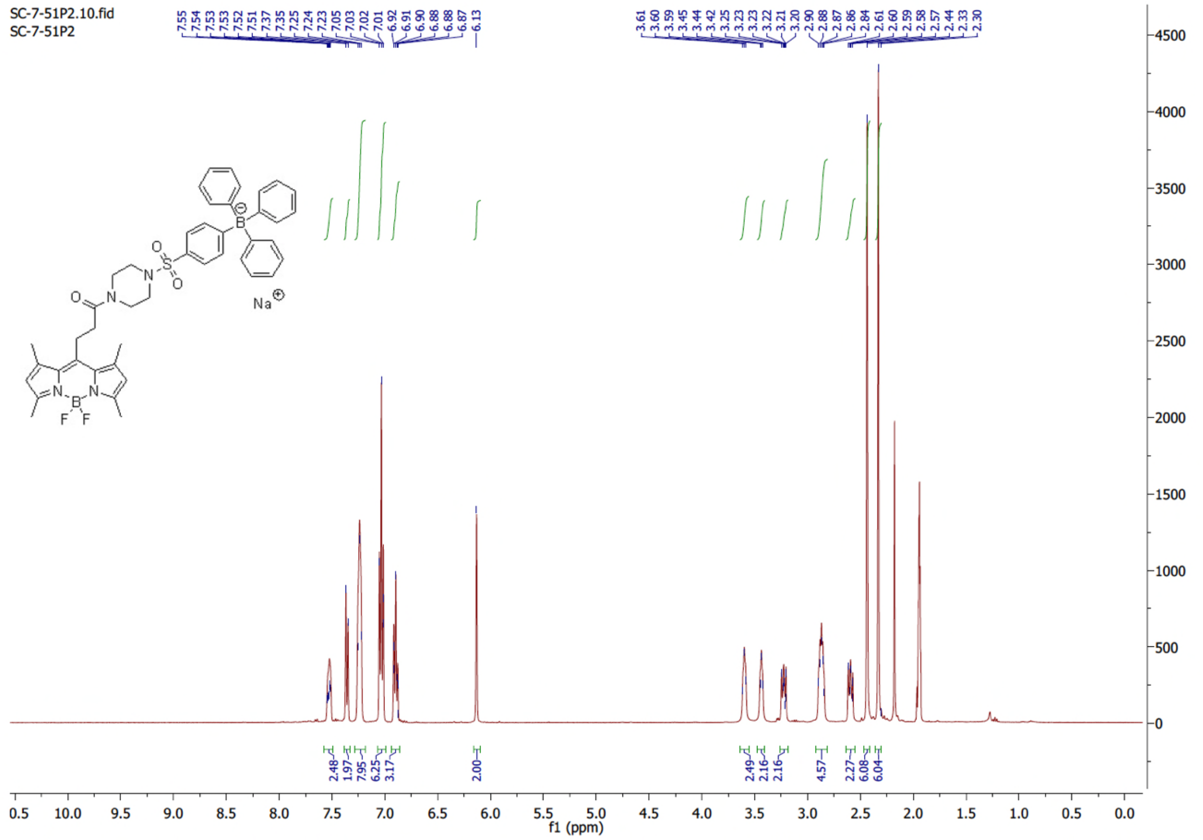

^{13}**C NMR Spectrum**

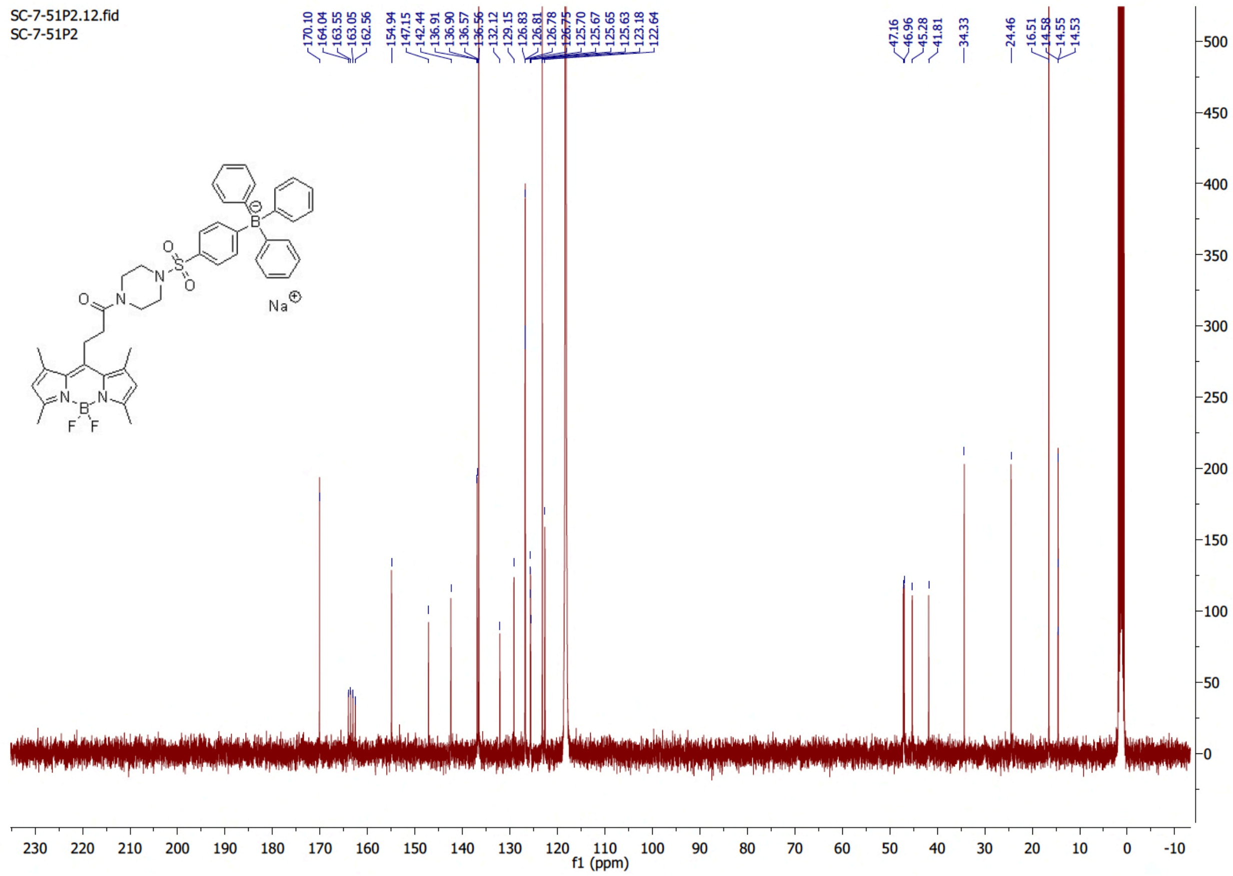

^{11}B NMR spectrum

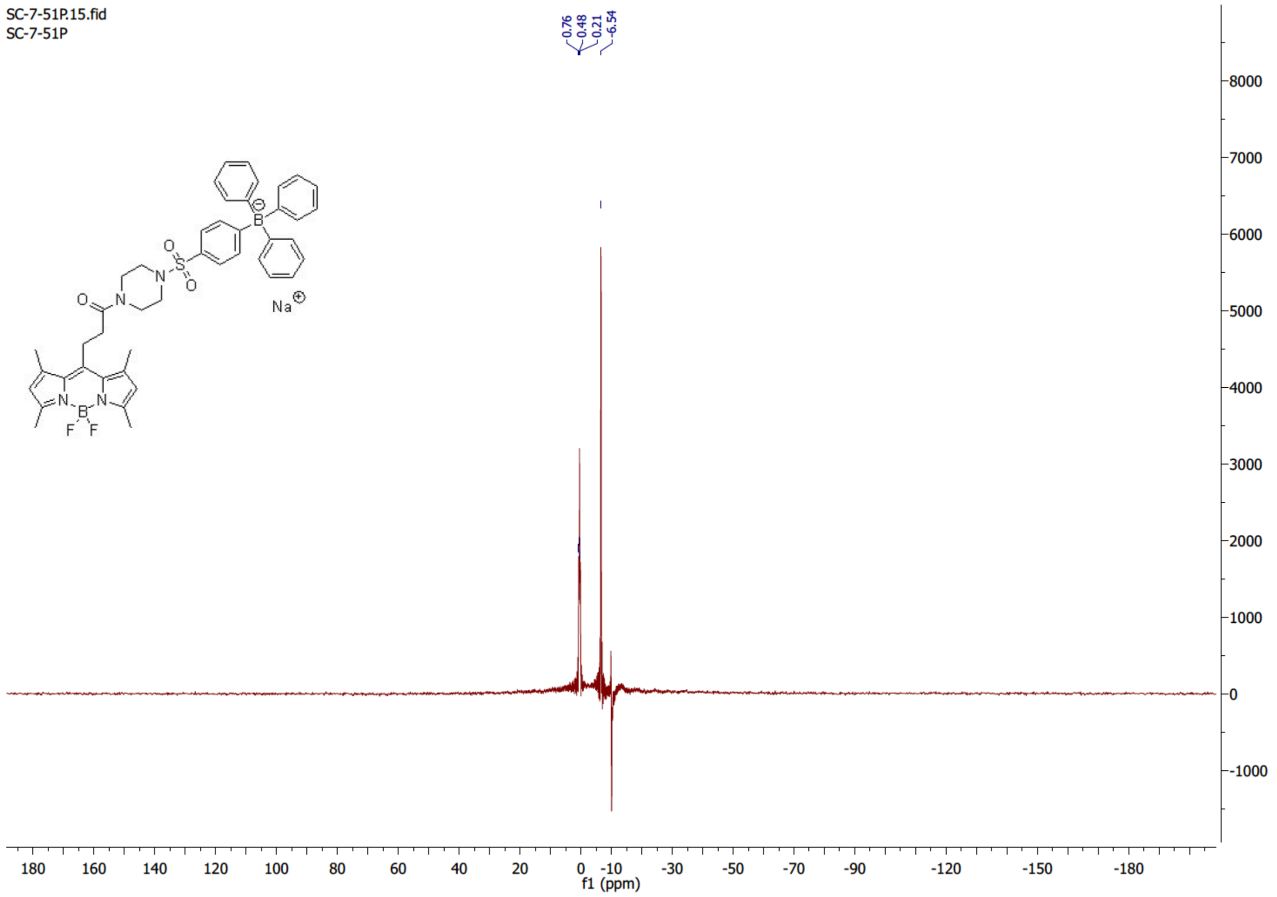

Sodium (4-{4’-[7”-(diethylamino)coumarin-3”-carbonyl]piperazin-1’-ylsulfonyl}phenyl)triphenylborate, TPBCoumarin (sodium salt).

^{1}H NMR Spectrum

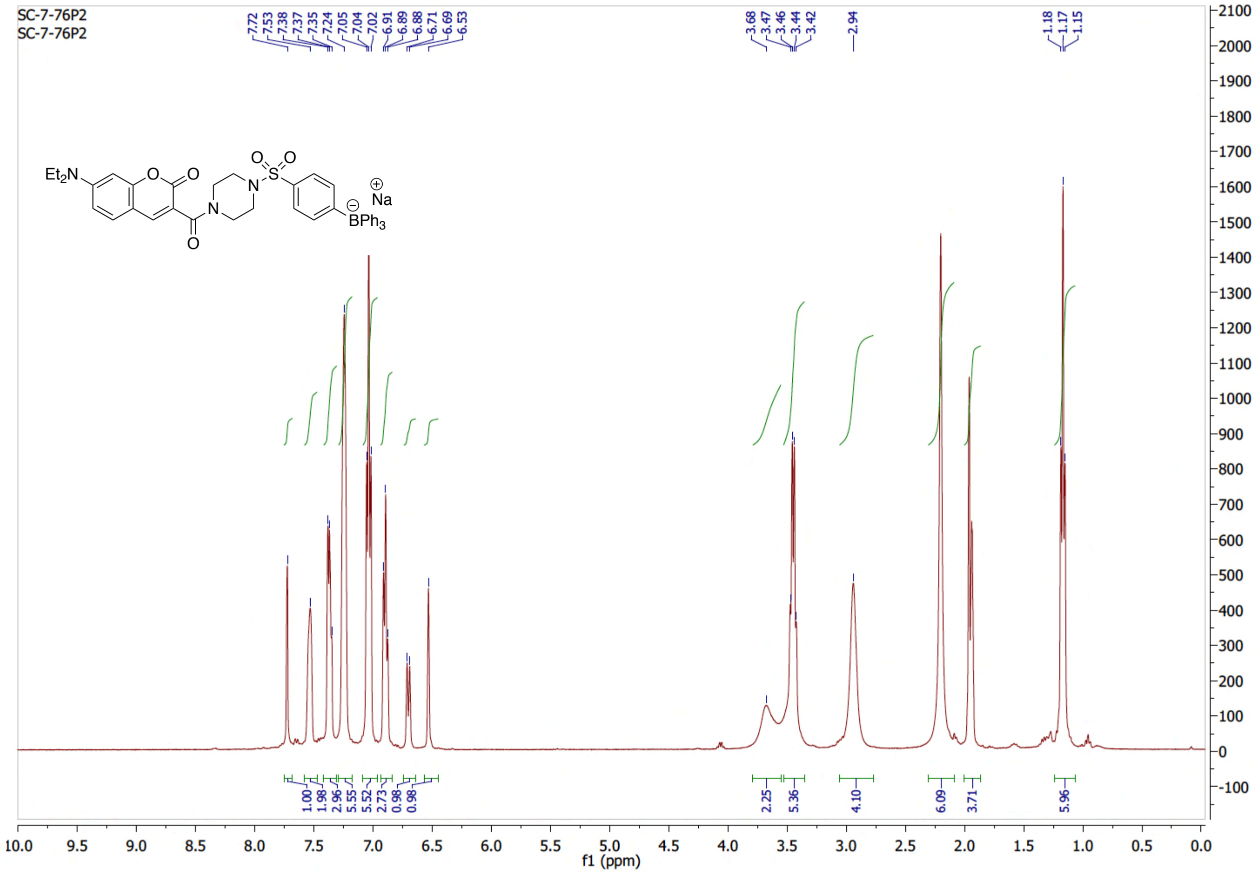

^{13}C NMR Spectrum

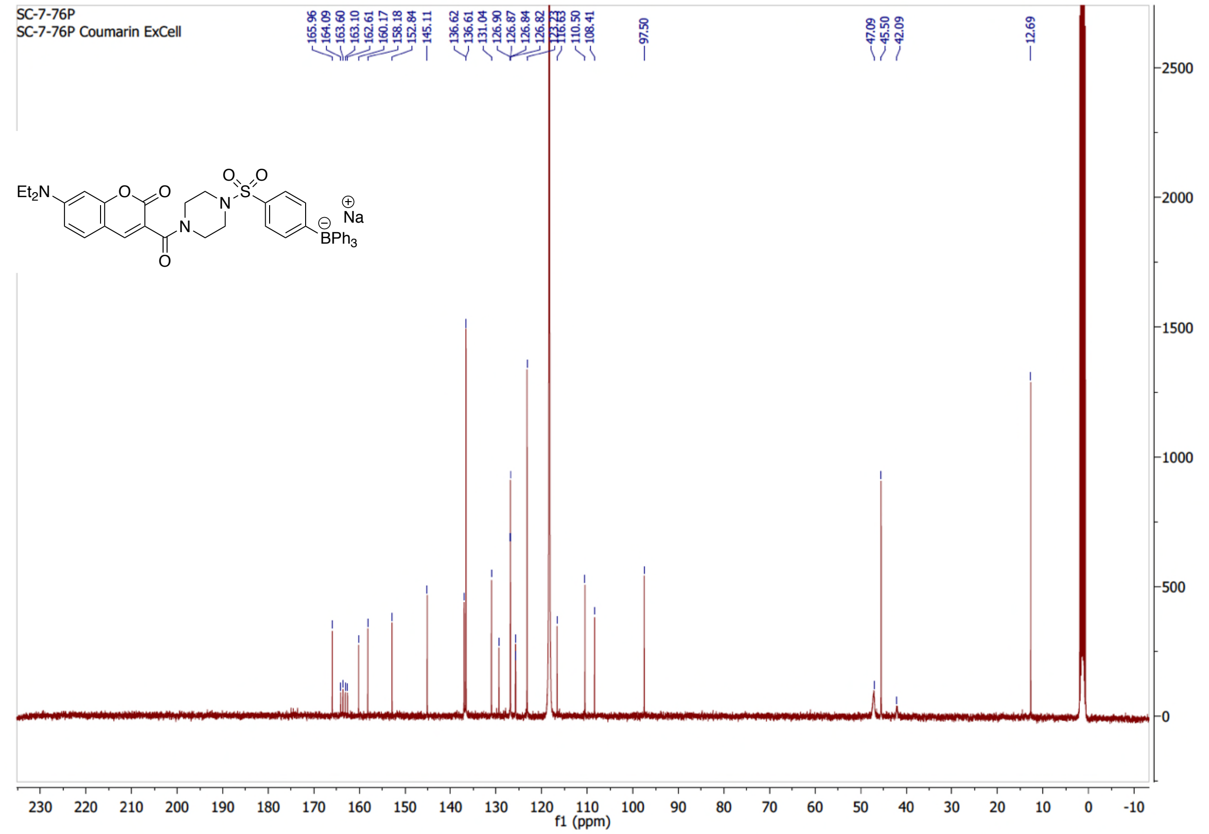

^{11}B NMR spectrum

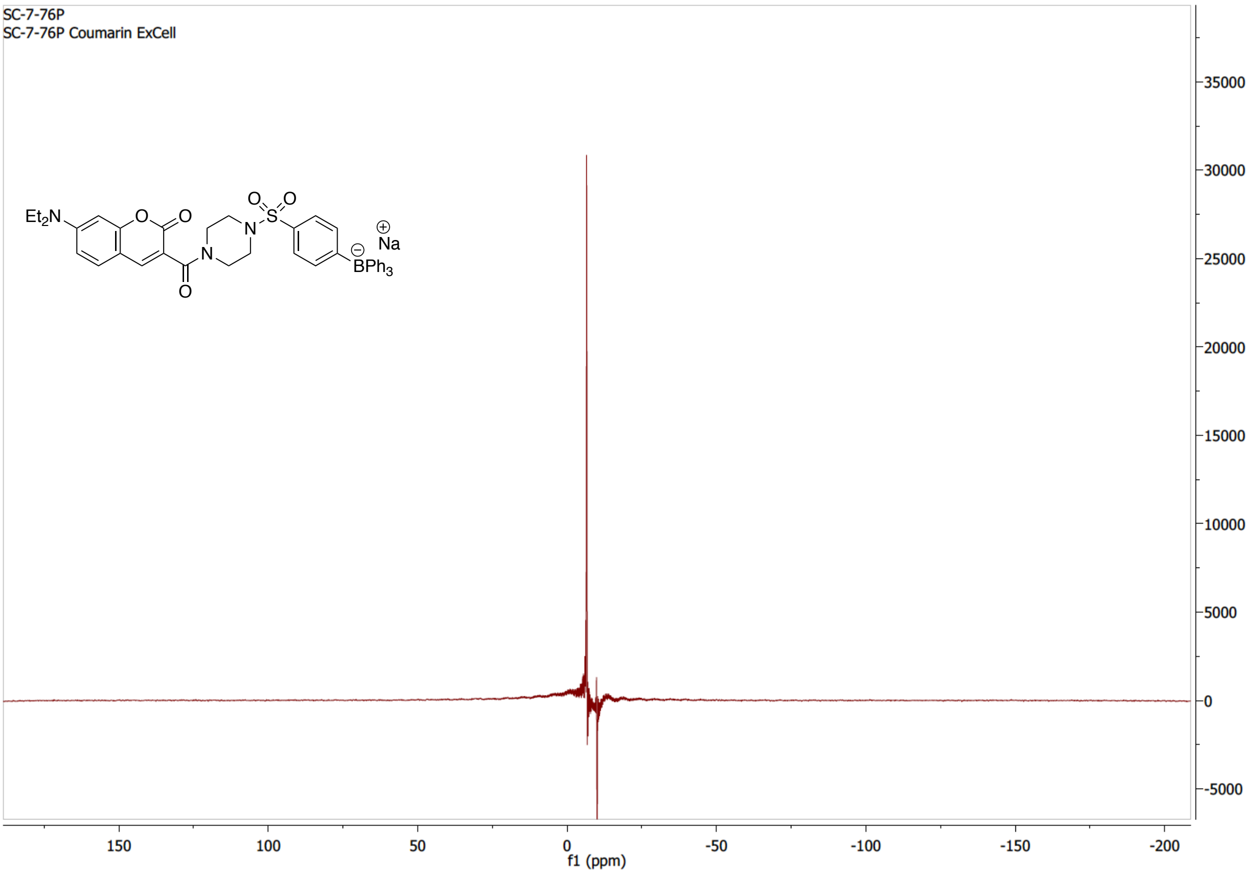

Supplement: Supplementary file 2 [file CC-057-D0CC07924C-s002.pdf]
